# Spatial architectures of somatic mutations in normal prostate, benign prostatic hyperplasia and coexisting prostate cancer

**DOI:** 10.1038/s12276-023-01140-8

**Published:** 2024-01-04

**Authors:** Jeesoo Chae, Seung-Hyun Jung, Eun Ji Choi, Jae Woong Kim, Na Yung Kim, Sung Won Moon, Ji Youl Lee, Yeun-Jun Chung, Sug Hyung Lee

**Affiliations:** 1 Department of Cancer Evolution Research Center, College of Medicine, The Catholic University of Korea, 06591 Seoul, South Korea; 2 Department of Biochemistry, College of Medicine, Ewha Womans University, 07804 Seoul, South Korea; 3 Department of Biochemistry, College of Medicine, The Catholic University of Korea, 06591 Seoul, South Korea; 4 Integrated Research Center for Genome Polymorphism, College of Medicine, The Catholic University of Korea, 06591 Seoul, South Korea; 5 Department of Biomedicine & Health Sciences, College of Medicine, The Catholic University of Korea, 06591 Seoul, South Korea; 6 Department of Pathology, College of Medicine, The Catholic University of Korea, 06591 Seoul, South Korea; 7 Department of Urology, College of Medicine, The Catholic University of Korea, 06591 Seoul, South Korea; 8 Department of Microbiology, College of Medicine, The Catholic University of Korea, 06591 Seoul, South Korea

**Keywords:** Medical genetics, Genetics research

## Abstract

This study aimed to identify somatic mutations in nontumor cells (NSMs) in normal prostate and benign prostatic hyperplasia (BPH) and to determine their relatedness to prostate cancer (PCA). From 22 PCA patients, two prostates were sampled for 3-dimensional mapping (50 normal, 46 BPH and 1 PCA samples), and 20 prostates were trio-sampled (two normal or BPH samples and one PCA sample) and analyzed by whole-genome sequencing. Normal and BPH tissues harbored several driver NSMs and copy number alterations (CNAs), including in *FOXA1*, but the variations exhibited low incidence, rare recurrence, and rare overlap with PCAs. CNAs, structural variants, and mutation signatures were similar between normal and BPH samples, while BPHs harbored a higher mutation burden, shorter telomere length, larger clone size, and more private NSMs than normal prostates. We identified peripheral-zonal dominance and right-side asymmetry in NSMs, but the asymmetry was heterogeneous between samples. In one normal prostate, private oncogenic RAS-signaling NSMs were detected, suggesting convergence in clonal maintenance. Early embryonic mutations exhibited two distinct distributions, characterized as layered and mixed patterns. Our study identified that the BPH genome differed from the normal prostate genome but was still closer to the normal genome than to the PCA genome, suggesting that BPH might be more related to aging or environmental stress than to tumorigenic processes.

## Decoding prostate cells: BPH more linked to age than cancer

The study explores the genetic differences between regular prostate cells, benign prostatic hyperplasia (non-cancerous enlargement of the prostate) cells, and prostate cancer cells. The researchers found that regular and benign prostatic hyperplasia cells are more alike than they are to prostate cancer cells. Using whole-genome sequencing, they discovered that benign prostatic hyperplasia cells had a higher rate of genetic changes, more clonal expansion, and more subclonal somatic mutations than regular cells. However, the difference was small, suggesting that benign prostatic hyperplasia is a non-cancerous disease. The study also found no link between somatic mutations and prostate cancer development. These findings offer valuable insights into the cell characteristics of benign prostatic hyperplasia and its connection with prostate cancer. Further studies are required to understand the clinical importance of these genetic features. This summary was initially drafted using artificial intelligence, then revised and fact-checked by the author.

## Introduction

As opposed to the earlier concept that nontumor cells do not harbor somatic mutations, somatic mutations in nontumor cells (NSMs) are known to occur in whole-body organs^1–3^. To date, research has shown that (i) the NSM prevalence is different depending on the organ; (ii) NSM number and clone size increase with age, inflammation, and mutagen exposure; (iii) although most NSMs are not cancer drivers, over 140 somatic driver NSMs have been identified across organs; (iv) mutation signatures of NSMs are commonly related to aging, but other signatures have been reported; and (iv) the causal relationship between NSMs and cancer risk remains uncharacterized^1,4–7^. NSMs include early embryonic mutations (EEMs) that accumulate throughout life after the first cell division^2,8–12^. An earlier study showed that prostate NSMs accumulated over a lifetime with two large waves during embryogenesis and puberty and were enriched in peripheral areas compared to periurethral areas^13^. However, somatic drivers, rearrangements, and copy number alterations (CNAs) among prostate NSMs are rare^13^. Even with this discovery, the NSMs of benign prostatic hyperplasia (BPH) and their relatedness to prostate cancer (PCA) have not been studied. Since NSMs in nonneoplastic diseases are common in other organs (both pro- and anti-disease development)^5,14–16^, simultaneous analysis of normal and BPH NSMs is essential to understand normal prostate progression to BPH or PCA.

Detection methods for NSMs vary depending on the study’s purposes. Whole-genome sequencing (WGS) of single-cell derived cultures allows NSM detection in single genomes but does not provide spatial information in the tissue or specific nonneoplastic states such as BPH^8^. Laser-capture microdissection provides NSMs with spatial information but can analyze a limited number of regions with small cell numbers and thus only a part of a tissue^9,17^. Deep-targeted sequencing of a grid of samples covers a larger area, but the accuracy of spatial histology is lower^4^.

Our study aimed to analyze NSMs in large areas with respect to the tissue location and coexisting lesions (BPH or PCA). To this end, we adopted a hybrid design in which WGS results of serial microdissections of prostate glands obatined through mechanical microdissection across multiple areas were compared^18^. We analyzed two prostates with extensive 3-dimensional (3D) sampling and 20 prostates with a trio of prostate samples (one tumor and two normal/BPH glands), thus controlling for both intraprostate and interprostate differences. Using these samples, we investigated the following questions: (i) genomic differences and relatedness of normal, BPH, and PCA genomes and (ii) identification of normal and BPH genomic features with biological and clinical significance.

## Materials and methods

### Sample collection and pathology examination

**Fig. 1:**
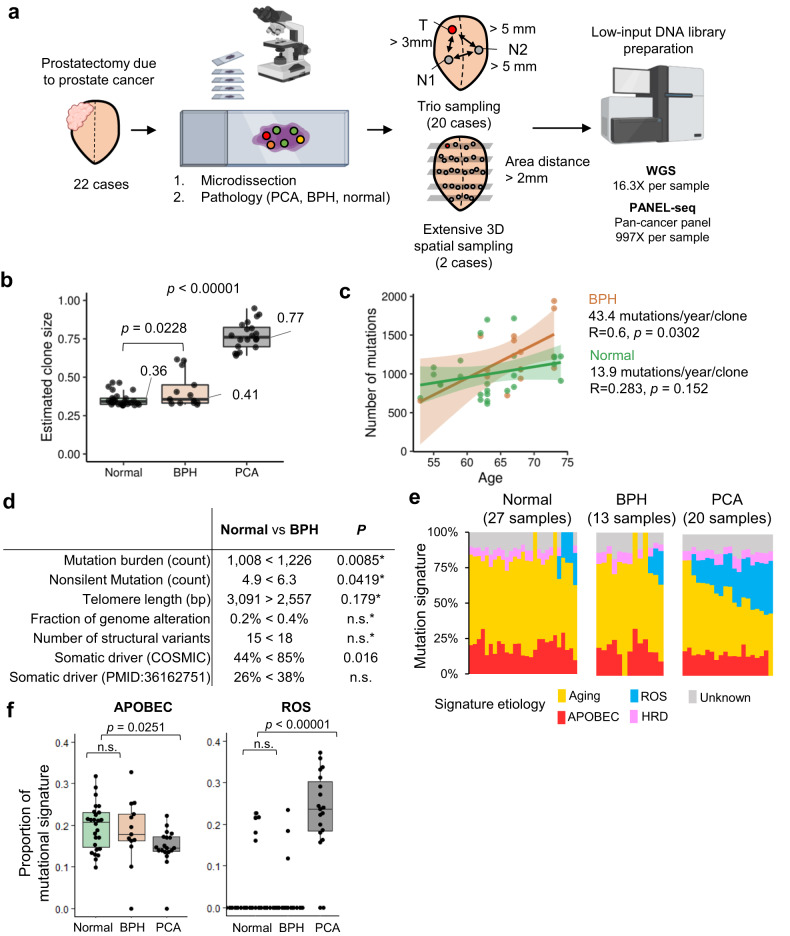
Study design and age-mutation correlations in normal and BPH samples. **a** Graphical summary showing 22 prostate tissues (20 cases with trio sampling and two cases with extensive 3D spatial sampling), followed by microdissection and low-input DNA whole-genome sequencing (WGS) and deep-panel sequencing. **b** Clone sizes, estimated from the peak of the variant allele frequency (VAF) in normal, BPH, and PCA samples. **c** The mutation burden increased with age in both normal prostate and BPH clones (shaded area: 95% confidence interval). The regression model for the normal tissue shows a slope of 16 mutations per year per clone with an *R*^2^ of 0.10. The slope of BPH samples (43.4 mutations/year/clone with an *R*^2^ of 0.36) was higher than that of normal samples. **d** Summary of genomic profiles between normal and BPH samples. Asterisk (*) indicates age-corrected regression *p* value. **e** Aging, APOBEC, ROS, and HRD mutational signatures in normal, BPH, and prostate cancer samples. **f** Comparison of mutational signature proportions between normal, BPH, and prostate cancer samples. n.s., *p* > 0.05.

Frozen radical prostatectomy tissues of unifocal PCAs from 22 patients without prior chemotherapy before surgery were obtained from the Korea Prostate Bank (Seoul, Korea) with institutional review board approval from the Catholic University of Korea (MC20TISI0098) (Supplementary Table 1). The tissues used were collected by the ‘alternative slices mirror image’ method^19^ and consisted of 8–14 blocks (approximately half of a prostate) per case that were mapped to vertical and horizontal locations (Supplementary Fig. 1). The gland epithelial cells were manually microdissected (157 areas: 77 normal, 59 BPH, and 21 PCA) from 9 serial sections per microdissection area with distant margins, collecting 5000–10,000 cells for each area (Fig. 1a and Supplementary Fig. 1)^18^. For the samples PCA-28 and PCA-49, 58 widespread areas (1 tumor, 46 BPH, and 11 normal areas) and 39 areas (39 normal), respectively, were 3D-sampled. For another 20 cases (20 tumor, 13 BPH, and 27 normal areas), we used trio sampling for each case with distant margins (one normal or BPH (N1) sample and one tumor (T) sample on the ipsilateral side and another normal or BPH sample on the contralateral side (N2)) (Fig. 1a). Further information is included in the Online Methods. Additionally, we collected 10 BPH tissue samples from individuals without PCA to compare the somatic profiles of pure BPH with BPH accompanying PCA.

### WGS data generation

DNA library preparation of microdissected tissue samples was performed as previously described using a low-input enzymatic fragmentation-based library preparation method^20^. In brief, DNA samples (20 μl) were mixed with 50 μl Ampure XP beads (Beckman Coulter Corporation, Miami, FL) and 50 μl TE buffer (Thermo Fisher Scientific, Waltham, MA) at room temperature. After the binding reaction and magnetic bead separation, genomic DNA was washed twice with 75% ethanol. Each sample was mixed with 7 μl 5X Ultra II FS buffer and 2 μl Ultra II FS enzyme (New England BioLabs, Ipswich, MA) and incubated on a thermal cycler for 12 minutes at 37 °C followed by 30 minutes at 65 °C. Following DNA fragmentation and A-tailing, each sample was incubated for 20 minutes at 20 °C with a mixture of ligation master mix and ligation enhancer (New England BioLabs), 0.9 μl of nuclease-free water (Thermo Fisher Scientific) and duplexed adapters (5’-ACACTCTTTCCCTACACGACGCTCTTCCGATC*T-3′, 5′-phos-GATCGGAAGAGCGGTTCAGCAGGAATGCCGAG-3′). Adapter-ligated libraries were purified using Ampure XP beads. After elution and bead separation, DNA libraries were amplified by PCR with KAPA HiFi Hot Start ReadyMix (Roche, Basel, Switzerland), PE1.0 primer (5′-AATGATACGGCGACCACCGAGATCTACACTCTTTCCCTACACGACGCTCTTCCGATC*T-3′), and iPCR-Tag (5’-CAAGCAGAAGACGGCATACGAGATXGAGATCGGTCTCGGCATTCCTGCTGAACCGCTCTTCCGATC-3′; ‘X’ represents unique 8-base indices). The sample was then mixed and thermally cycled as follows: 98 °C for 5 minutes; 7 cycles of 98 °C for 30 seconds, 65 °C for 30 seconds, and 72 °C for 1 minute; and finally 72 °C for 5 minutes. Amplified libraries were purified using a 0.7:1 volumetric ratio of Ampure Beads to PCR product and eluted into 25 μl nuclease-free water. DNA libraries were assessed by Tapestation 4200 (Agilent Technologies, Santa Clara, CA) and were sequenced on an Illumina HiSeq X platform (Illumina, San Diego, CA) according to the manufacturer’s instructions.

### WGS data processing

Sequenced reads were aligned to the human reference genome (GRCh38) using BWA-mem v0.7.17. Aligned reads were sorted, deduplicated with Picard (available at http://broadinstitute.github.io/picard), and locally realigned using GATK v4.1.6.0^21^. Telomerecat^22^ was used to estimate the length of telomeres in each sample. We utilized Sequenza *v3.0*^23^. to estimate the tumor purity, ploidy, and somatic copy number profiling. The Sequenza software automatically selected the optimal purity and ploidy values, which were manually corrected for several cases through refinement. We estimated the degree of clonality of each nonneoplastic clone by taking the median cell fraction of the clonal mutations of passed base substitution calls.

### Single-base substitution and indel analysis

Somatic mutations were identified by Mutect2 v.2.2.0, selecting biallelic and ‘PASS’ mutations. Mutations with a variant allele frequency (VAF) greater than 0.01 in the population database were removed. Single-base substitutions (SBSs) were analyzed with a modified application of Ellis et al.^24^. Germline variants were filtered out for two multiregion sampling cases (PCA-28 and PCA-49) using an exact binomial distribution test. Artifactual variant filtering was performed with a beta-binomial test and cross-sample genotyping. Fragment-based statistics were calculated using ‘AnnotateBAMStatistics’ of SangerLCMFiltering. Mutations expected to be germline-shared in all trio samples with an average VAF > 0.3 were removed. Indels having fewer than seven total reads or two variant reads in a sample or mapping to paralogous genomic regions were removed. The final set of mutations was annotated with ANNOVAR^25^ to assess functional impact and used for phylogenetic tree construction with the maximum likelihood algorithm^26^. The clonal status of each mutation was estimated by calculating the mutant copy number^27^.

### Mutational signature analysis

Using the SIGNAL mutational signature reconstruction tool^28^, we decomposed the mutational signatures of each sample. The relative contribution of signatures was calculated by refitting seven consensus mutational signatures (SBS1, 2, 3, 5, 13, 18, 92), a panel of prostate-associated signatures declared in SIGNAL.

### Identification of ancestral mutations in spatial sequencing

Mosaic mutations with high allele frequency resembling germline variants were detected in a subset of samples during extensive spatial sequencing. These mosaic mutations shared by more than two clones were classified as early mutations occurring in a lifetime after validation with SAMtools mpileup and manual inspection with the Integrative Genomics Viewer (IGV).

### Statistical analysis

We performed statistical analyses using R version 3.6.3, including linear regression, Kruskal‒Wallis, Mann‒Whitney *U*, and chi-square tests.

## Results

### Genomic landscapes of normal and BPH tissues

From microdissected cells, we generated WGS and deep-panel sequencing data with average depths of 16.3 and 997, respectively (Fig. 1a, Supplementary Figs. 2–5 and Supplementary Tables 2–9). To exclude bias from single cases’ multiple samples (PCA-28 and PCA-49), we analyzed the genomic landscapes of normal and BPH tissue in the trio samples of 20 patients. The clone size of BPH tissues (0.41) estimated from the VAF was larger than that of normal tissues (0.36) (*p* = 0.023) and that of PCA tissues (0.77) was far larger than those of normal and BPH tissues (*p* = 1.09 × 10^8^, Fig. 1b). The linear correlation of age and NSMs in BPH was stronger than that in the normal prostate (Fig. 1c). We found similar mutation burdens and telomere lengths in normal prostate glands between previous studies and ours (1111 in Moore et al. and 1008 in our study, *p* > 0.05, Supplementary Fig. 2). Of note, we observed a higher mutation burden and shorter telomere length in BPHs than in normal prostates. However, no significant differences in CNAs and structural variants were observed between them (Fig. 1d). A total of 136 nonsilent NSMs were identified in normal samples, with an average of 4.9, and 81 NSMs were identified in BPHs, with an average of 6.3, in the trio cases (*p* = 0.042), including the COSMIC cancer genes *FOXA1, KMT2C*, and *BCOR* (Supplementary Table 7). Between normal and BPH samples, there was a significant difference in the proportions of the COSMIC cancer driver genes (*p* = 0.0161) but not of NSM driver genes^1^ (*p* = 0.0561) (Fig. 1d). Of the prostate NSM drivers detected in a previous study^13^, *FOXA1* and *KMT2A* were identified in our NSMs. However, none of the driver NSMs detected in the trio samples were shared with PCA. Large CNAs were detected in four normal samples, but none were detected in BPH samples (*p* > 0.05). In PCA samples, mutation burden, CNAs, structural variations, COSMIC drivers, and chromosomal abnormalities were significantly higher than in nonneoplastic prostates (normal and BPH).

We examined the correlation between clinical data (tumor driver type, PSA, Gleason score, and tumor size) and mutation burdens (Supplementary Fig. 6 and Supplementary Table 10). The somatic mutation burden of PCAs showed a significant positive correlation with Gleason score (*R* = 0.701 and *p* = 0.0006) and a differential pattern by tumor drivers. However, these clinical data points lacked a notable correlation with NSMs.

We compared the genomic profiles of BPH coexisting with PCA to those of BPH without accompanying PCA to determine if these features changed with the presence of PCA (Supplementary Fig. 7 and Supplementary Tables 11–12). Whole genome analysis of the pure BPH samples (*n* = 10) indicated comparable genomic profiles with those of the BPH samples accompanying PCA. Both groups exhibited a pronounced correlation between mutation burden and age and between mutation burden and telomere length. Furthermore, the frequency of somatic drivers was similar across both groups. This highlights the underlying genomic similarities between pure BPH and BPH coexisting with PCA.

### Mutation signatures of normal and BPH tissues

Aging signatures were the most common mutation signatures in normal, BPH, and PCA samples (Fig. 1e). The number of cases with a reactive oxygen species (ROS) signature was significantly higher in PCA samples (90%) than in normal (22%) and BPH (23%) samples (*p* < 0.00001). There was no significant difference in ROS, APOBEC, or homologous recombination deficiency (HRD) signatures between normal and BPH samples (*p* > 0.05) (Fig. 1f).

### Subclonal architectures of normal and BPH tissues

**Fig. 2:**
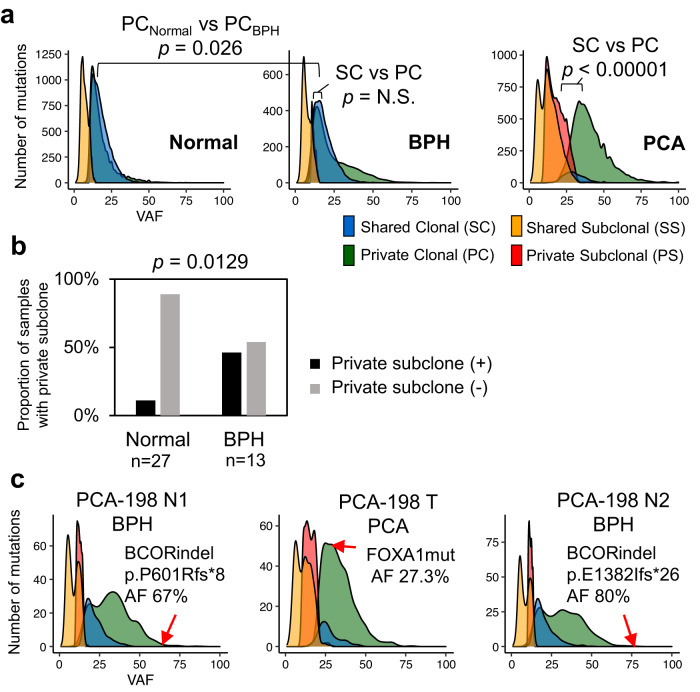
Subclonal genomic architectures of normal prostate and BPH. **a** Variant allele frequency (VAF) distribution grouped by the mutation clusters. BPH shows a higher VAF of PC mutations than normal tissues (*p* = 0.026). **b** Proportion of PS mutations in normal and BPH samples. BPH showed a higher PS proportion than normal tissues (chi-square *p* = 0.0129). **c** Example of private clonal cell cluster expansion in BPHs. In PCA-198, both BPHs (N1 and N2) show PC expansion with different BCOR indels.

To determine the subclonal genomic architectures of normal and BPH tissues, we categorized the NSMs into shared clonal (SC), private clonal (PC), shared subclonal (SS), and private subclonal (PS) types (Fig. 2a and Supplementary Fig. 8). All four types were highly enriched in PCA samples compared to normal and BPH samples. BPH tissues harbored more PC mutations than normal tissues, with an average of 525 mutations in BPH and 397 in normal (*t* test *p* = 0.100) and average VAFs of 21.1% and 17.0%, respectively (*p* = 0.026). In addition, PS mutations were more frequent in BPH patients than in normal controls (*p* = 0.0129, Fig. 2b). Although no NSMs were shared in the same prostates, one prostate (PCA-198) showed different *BCOR* indels in two BPH samples (Fig. 2c and Supplementary Fig. 9), suggesting field effects of this gene in this case. In phylogenetic analysis, most PCA mutations exhibited early divergence from the trunks (Supplementary Fig. 10). Furthermore, ROS signatures were predominant in PC mutations in PCA, found in nearly 94% of the clusters. In addition, ROS were virtually absent in SC mutations (Supplementary Fig. 11). This emphasizes distinct molecular dynamics and suggests that oxidative stress-related mutations might predominantly emerge during individual tumor progression rather than being a foundational event.

### Spatial genome mapping of the two prostates

**Fig. 3:**
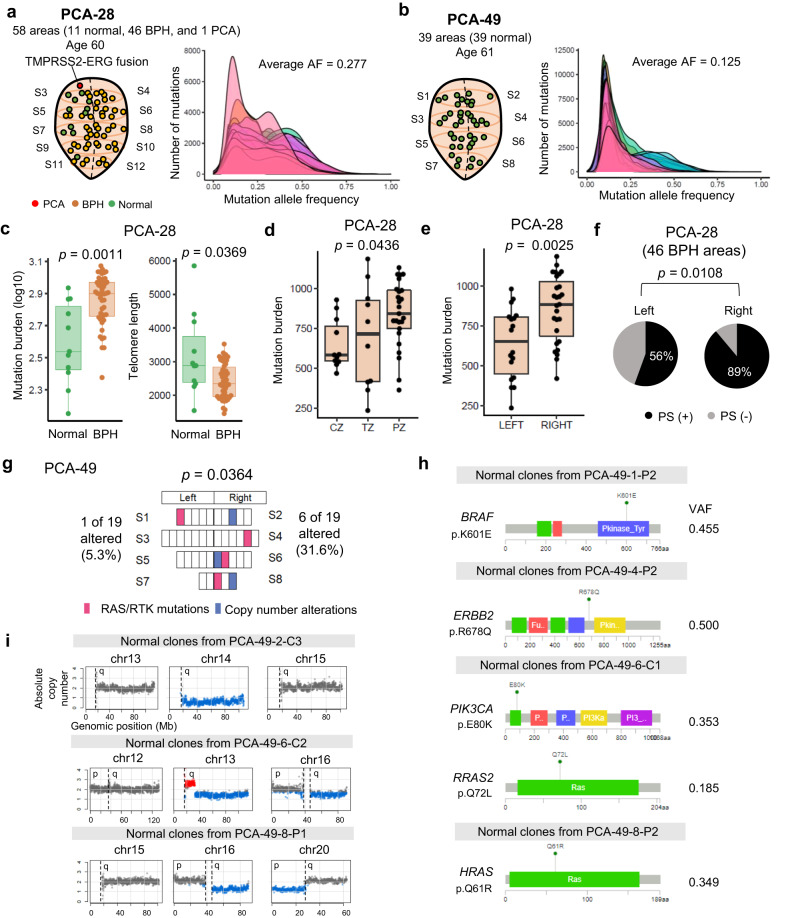
Spatial sequencing reveals intraprostate genomic heterogeneity. **a**, **b** Sampling information and VAF distribution of PCA-28 (**a**) and PCA-49 (**b**). **c**–**f** BPHs in PCA-28 show significantly higher mutation burden and shorter telomere length than normal cells. Green: normal; Brown: BPH (**c**). Significant differences in mutation burden by zonal distribution (**d**) (H-test *p* = 0.0436) and by left-right sidedness (**e**) (*p* = 0.0025). **f** Proportions of private subclones in left and right BPHs (chi-square *p* = 0.0108). **g** Somatic alterations of PCA-49. The right side has significantly more somatic alterations (31.6%) than the left side (5.3%, *p* = 0.0364). **h** Distribution of convergent RTK-RAS pathway mutations in PCA-49. **i** Large-scale chromosomal alterations in PCA-49. Red: gain. Blue: loss.

We conducted 3D spatial WGS in two prostates (PCA-28 and PCA-49) to identify intraprostate genomic heterogeneity (Fig. 3a, b, and Supplementary Fig. 12). In PCA-28, BPHs showed a higher mutation burden and shorter telomere length than normal tissues (Fig. 3c). Among the three prostate zones (peripheral zone (PZ), central zone (CZ), and transitional zone (TZ)), the mutation burdens of BPHs were different (H-test *p* = 0.0436), with the highest in the PZ and the lowest in the TZ (Fig. 3d), consistent with previous data showing enrichment in the peripheral area^13^. We compared the NSMs between the left vs. right, anterior vs. posterior, and upper vs. lower dimensions (Supplementary Fig. 13). In PCA-28, we observed asymmetric BPH genomes with higher mutation burden, lower telomere length, and higher PS proportion on the right side (*p* = 0.0025, *p* = 0.0181, and *p* = 0.0188, respectively, Fig. 3e, f).

In PCA-49, the asymmetry of mutation burden and telomere length identified in PCA-28 were not observed. However, this prostate showed asymmetry of receptor tyrosine kinase (RTK)/RAS mutations and large CNAs (*p* = 0.0364, Fig. 3g). Of note, all RTK/RAS NSMs (*BRAF, ERBB2, PIK3CA, HRAS*, and *RRAS2*) were singleton mutations with relatively high VAFs (18.5–50.0%) in different normal areas (Fig. 3h), which was confirmed by panel sequencing. Although these mutations had been reported as cancer genes, all except *HRAS* p.Q61 were uncommon mutations. In PCA-49, large-scale CNAs were evident in three normal areas (Fig. 3i). 16q losses were recurrent in two normal areas, but the lineages differed. We analyzed RTK/RAS mutations in the trio prostates and found that 4% of normal, 31% of BPH, and 25% of PCA regions harbored RAS/RTK mutations (Supplementary Fig. 14). RAS/RTK NSMs were identified in 7% of PCA-28. In the upper-lower and anterior-posterior dimensions, only PCA-49 showed lower asymmetry in the mutation burden (*p* = 0.025, Supplementary Fig. 13).

To address the possible relatedness of NSMs to PCA development, we analyzed the NSM distribution between an area close to the tumor (PCA-ipsilateral, normal and BPH, T and N1 distance: >2 mm) and an area away from the tumor (PCA-contralateral, normal and BPH, T and N2 distance: >5 mm). We observed no significant difference in the mutation burdens and telomere lengths (Supplementary Fig. 15).

### EEMs in prostates

**Fig. 4:**
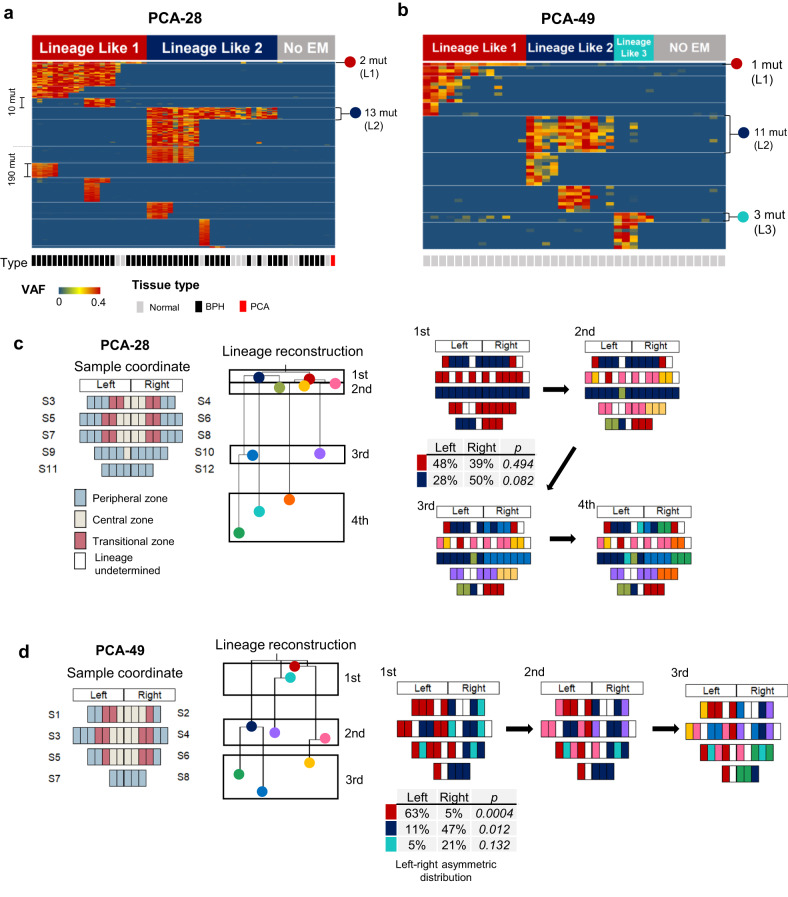
Early embryonic mutations in the prostate and reconstruction of ancestral lineages. **a**, **b** Pattern and allele frequency of early embryonic mutations in PCA-28 (**a**) and PCA-49 (**b**) with the disease types displayed in the bottom plots. PCA-28 was divided into three groups: lineage-like 1, lineage-like 2, and early mutation-absent; PCA-49 was divided into four groups: lineage-like 1, 2, and 3 and early mutation-absent. **c**, **d** Graphical distribution, lineage reconstruction, and lineage progression by color-coded representation of the ancestral mutations in PCA-28 (**c**) and PCA-49 cases (**d**). Each unit of the sample coordinate map represents a sample.

To explore the potential contribution of early ancestral mutations acquired during prostate development to the asymmetric somatic profiles in PCA-28 and PCA-49, we conducted an analysis of early ancestral mutations, including EEMs. The distributions of EEMs differed from each other (Fig. 4a, b). Few EEMs were detected in coding regions (0.67% in PCA-28 and 3.77% in PCA-49). The earliest ancestral EEMs of PCA-28 were two clones (L1 and L2) that branched three times to subsequent lineages (Fig. 4c). L1 and L2 were distributed on the vertical layers, maintaining vertically alternating lineage asymmetry (layered pattern, Fig. 4c). In PCA-49, the ancestral EEMs were mixed from the first generation on the same layers, which revealed right-left asymmetry of the lineage distributions (mixed pattern, Fig. 4d). Mutation burden, signatures, prostate zone, and histopathology (normal and BPH) showed no significant association with these patterns. The private oncogenic mutations shown in Fig. 3g were unrelated to the EEM pattern (*p* > 0.05, Fig. 4d).

## Discussion

Our study found that the normal and BPH genomes were more similar to each other and different from that of PCA. Both normal and BPH samples harbored NSMs and CNAs, including driver genes, but were far less common than PCA. No prostate NSMs were shared with PCA, and no proximity was identified between NSMs and PCA mutations. BPHs showed higher NSM burden, shorter telomere length, more clonal expansion, and more subclonal NSMs than normal prostates, but the genomic difference was minimal. These data suggest that BPHs have genomic alterations, but these alterations are found in quantitatively and qualitatively naive genomes that may not be directly related to PCA development. However, some cancer-related features were found unexpectedly; for example, convergent RTK/RAS NSMs in the normal prostate need to be identified to determine biological and clinical significance.

Previous studies^13,29,30^ identified that normal and BPH genomes harbored no evidence of driver genomic alterations related to carcinogenesis, with low coding mutation rates, minimal CNAs, and no genomic rearrangements^13,31^. Expanding upon these findings, our comparative analysis of BPHs with corresponding normal cells revealed that BPHs had larger clone sizes, more private subclones, and more mutations in somatic driver genes than normal prostates. Furthermore, consistent with observations in normal prostatic epithelium^13^, we identified an age-related increase in mutational burden in BPH that surpasses that of normal cells. Conflicting observations on telomere length in BPH have been reported, comparing normal and tumor tissues^22,23^. However, our data suggest that BPHs occupy an intermediate position, possessing telomeres that are shorter than those in normal prostate cells but longer than those in tumor cells.

Our findings regarding the clonal structure and expansion of private clones/subclones further support the observation by Middleton et al., who reported BPH-specific somatic mutations and suggested cell population enrichment in 18 BPH tissues^21^. Our whole-genome-level profiling could provide a better understanding of the cellular characteristics of BPH.

The pathogenic significance of somatic mutations in BPHs is uncertain. However, *FOXA1*, a well-known PCA driver gene^32,33^, has been identified in previous studies^13^ as well as ours, suggesting its role as an early PCA driver or a caregiver for nonneoplastic prostate clones.

Through the high-resolution mapping of an entire prostate (PCA-49), we found a unique distribution of convergent NSMs in normal histologic areas. First, RTK/RAS gene mutations revealed high VAFs and consisted of uncommon variants. Second, they were singleton mutations detected in only one area. Third, each clone had no driver genes other than the RTK/RAS mutations. The data suggest that convergent RTK/RAS NSMs could constitute a cooperatively permissive environment in the entire prostate. This PCA patient was a 60-year-old Korean patient with Stage II PCA (Gleason 4 + 3, PSA 2.35, 0.6 cm in diameter), but no particular clinical features were identified. The relationship between the PCA genome and normal genomes of this prostate was not analyzed because of the nonavailability of the tumor tissue in the biobank. Further studies are needed to identify whether the convergent NSMs of a specific pathway are a general phenomenon in prostates. While our study identified convergent RTK/RAS mutations in PCA-49, it is essential to note that this observation stemmed from a single case. As a result, the significance of the RTK/RAS mutations in this case could be incidental. Further studies with a larger cohort and diverse sample sets will be required to validate this and ascertain its potential implications for PCA biology.

The SBS18 ROS mutation signature was enriched in PCAs compared to nonneoplastic prostates (Fig. 1e). ROS can damage DNA associated with the ROS mutational signature, which is a frequent signature in human cancers, including PCA^28,34^. An earlier BPH genomic study identified SBS18 in 28% of BPHs^29^, similar to the rates found in the normal (22%) and BPH (23%) tissues of the trio samples. The similar signatures between normal and BPH samples suggest that the mutagenic stimuli might not be qualitatively different.

Chronic inflammation produces cellular stimuli for NSM development and clonal expansion, as identified in liver cirrhosis, inflammatory bowel disease, and menstrual endometrium^16,35,36^. Bona fide features of BPH are hormonal imbalance and inflammation, resulting in prostate cell proliferation and chronic inflammatory cell infiltration. However, normal and BPH samples did not show a striking difference in overall genomic landscapes, suggesting that hormonal imbalance and inflammation might only be gentle mutagens for nonneoplastic prostates. High clonal expansions in liver cirrhosis, inflammatory bowel disease, and the menstrual endometrium involve continuous cycles of destruction and repair of parenchymal epithelial cells by chronic organ inflammation^16,35,36^. However, BPH does not exhibit continuous destruction and repair cycles, which could be the background for the less aggressive NSMs in BPH.

In the present study, we analyzed 3D asymmetry in two prostates, revealing right-left asymmetry in NSMs. In detail, however, the contents of genomic asymmetry were variable. In one case, the NSMs in BPH showed right side-propensity in mutation burden and telomere length. The other case exhibited right-side asymmetry in CNAs and oncogenic NSMs. Our data suggested that each prostate might have geometrically uneven mutagenic stimuli, resulting in a random NSM asymmetry. In earlier studies, later-generation EEMs became enriched in specific organs with asymmetry^9,11^. We further specified the enrichment patterns in two prostates, i.e., vertically layered and mixed distributions, indicating that the asymmetry and the distribution pattern might be highly variable among prostates. Furthermore, our study highlighted divergent EEM patterns in early embryogenesis. While the emergence of the prostate gland from the urogenital sinus by the 9th week is well documented, the specific impact of these early mutation patterns remains elusive. Efforts to correlate EEM patterns with somatic profiles, such as BPH status and overall mutation burden, found no definitive associations. Given the current knowledge, the role of these early mutations in disease and cancer predisposition warrants further investigation.

To find the proximity enrichment of NSMs to PCA, we analyzed areas close to and away from the tumor, but no proximity relatedness was found, suggesting that the geometric enrichment of prostate NSMs might not be directly related to PCA development.

Our genome data show that normal and BPH genomes are similar to each other and different from the PCA genome, supporting the theory that BPH is a nonneoplastic disease. BPHs showed naïve genomes but harbored genomic features of increased mutation burden and convergent NSMs, the clinical significance of which should be further studied.

### Supplementary information

Supplementary information

Supplementary Table 12

Supplementary Table 11

Supplementary Table 10

Supplementary Table 9

Supplementary Table 8

Supplementary Table 7

Supplementary Table 6

Supplementary Table 5

Supplementary Table 4

Supplementary Table 3

Supplementary Table 2

Supplementary Table 1

## Data Availability

The WGS dataset generated and/or analyzed during the current study will be publicly available at the NCBI SRA repository (PRJNA985804).
