# Supplementary material for: Spatial architectures of somatic mutations in normal prostate, benign prostatic hyperplasia and coexisting prostate cancer
Source: Exp Mol Med. 2024 Jan 4;56(1):168–76. doi: 10.1038/s12276-023-01140-8 (PMC10834420; doi:10.1038/s12276-023-01140-8)
Supplement: Supplementary file 1 — Supplementary information [file 12276_2023_1140_MOESM1_ESM.pdf]

This PDF file includes:

- **Online Methods**
- **Supplementary Fig. 1 to 15**
- **Legends for Supplementary Table 1 to 12**

Online Methods

Tissue specimen

For two PCAs, gland epithelial cells from 58 areas (1 tumor, 46 BPH, and 11 normal areas) of one patient (PCA-28) and 39 normal areas of the other patient (PCA-49) were microdissected by a pathologist, respectively (fig. S1). We did not analyze the tumor of the latter case because the tumor area was not available in the biobank blocks. These microdissection areas were widespread through the zones (PZ, transition zone (TZ), and central zone (CZ)) and the anatomical positions (horizontal and vertical). The distance between any of the two areas was at least 0.2 mm in length. Fresh frozen tissues were cut at 30 um thickness and stained with hematoxylin for 10 seconds without any fixation. Provided that the epithelial cells in a close distance are affected by similar mutagenic stimuli, we arbitrarily defined the unit of a microdissection area for a continuous thickness and width of epithelial cells under microscope. For this, gland epithelial cells per microdissection were procured from nine serial sections by a manually controlled microdissection under the microscope [1] that collected 5,000-10,000 cells for each area.

For another 20 cases (20 tumor, 13 BPH, and 27 normal areas), we analyzed epithelial cells from one normal or BPH (N1), and one tumor (T) areas at an ipsilateral side, and another normal or BPH area on the contralateral side (N2) (i.e., trio samples). The distances between N1 and T, N2 and T, and N1 and N2 were at least 2 mm, 5 mm, and 5 mm in length. The microdissected cells were overnight incubated in proteinase K-containing buffer and used for the WGS after heat inactivation that collected 5,000-10,000 cells for each area [1].

Panel sequencing data generation and processing

We performed targeted sequencing with DNA from microdissected tissue samples using OncoChase (ConnectaGen, Seoul, Korea) cancer panel. Sequencing libraries were generated using the Ion AmpliSeq Library Kit 2.0 (Thermo Fisher Scientific) and Ion Xpress barcode adapter kit (Thermo Fisher Scientific) according to the manufacturer's instructions. The sequencing libraries were normalized for templating on the Ion Chef (Thermo Fisher Scientific) and subsequently sequenced on the Ion S5 system (Thermo Fisher Scientific). Torrent Suite software version 5.12.1 (Thermo Fisher Scientific) was used to align raw sequence reads to the human genome (hg19) and detect genomic variants.

Somatic copy number analysis

For somatic CN alteration detection, we used the CN value of 2N data as a baseline for 1N and 1T samples, and 1N data as a baseline for SCNA detection in 1N. In the case where the 2N was PIN, we used 1N as a baseline for the analysis of 1T. In cases where multiple regions were sampled, we assigned one diploid genome sample as the baseline data.

Genomic rearrangements analysis

To identify initial somatic structural variants (SVs), we utilized Delly v2.0 [2]. We removed recurrent artifactual SV calls based on a panel of normal SV datasets established from non-neoplastic samples in this study. Discordant read pairs and soft-clipped reads were thoroughly reviewed by screening aligned reads in regions of interest from raw SV calls following the process of Park et al. [3]. We clustered SVs based on spatial proximity with cut-offs at an inter-breakpoint distance of < 5 Mb. A final SV cluster with more than 10 SVs in a clone was further classified into types of complex genomic rearrangements based on the criteria of Park et al. [3].

We manually reviewed several important SVs, such as ETS fusions and SV events in non-neoplastic clones, using IGV software [4]. Circos program was used to visualize SV clusters along with the CN profile of the 100kb window averaged coverage depth, with each SV cluster shown with different colors.

References

1 Lee, J. Y. *et al.* A simple, precise and economical microdissection technique for analysis of genomic DNA from archival tissue sections. *Virchows Arch* **433**, 305-309, doi:10.1007/s004280050253 (1998).

2 Rausch, T. *et al.* DELLY: structural variant discovery by integrated paired-end and split-read analysis. *Bioinformatics* **28**, i333-i339, doi:10.1093/bioinformatics/bts378 (2012).

3 Lee, J. J. *et al.* Tracing Oncogene Rearrangements in the Mutational History of Lung Adenocarcinoma. *Cell* **177**, 1842-1857 e1821, doi:10.1016/j.cell.2019.05.013 (2019).

4 Robinson, J. T. *et al.* Integrative genomics viewer. *Nat Biotechnol* **29**, 24-26, doi:10.1038/nbt.1754 (2011).

Supplementary Figure 1. Microdissection of normal epithelial cells of a prostate gland. a left column: a prostate gland lined by epithelial cells (red arrows in the box) and subepithelial connective tissue (black arrows). **b** The epithelial cells are partially detached by microdissection (red arrows) leaving the connective tissue (black arrows).

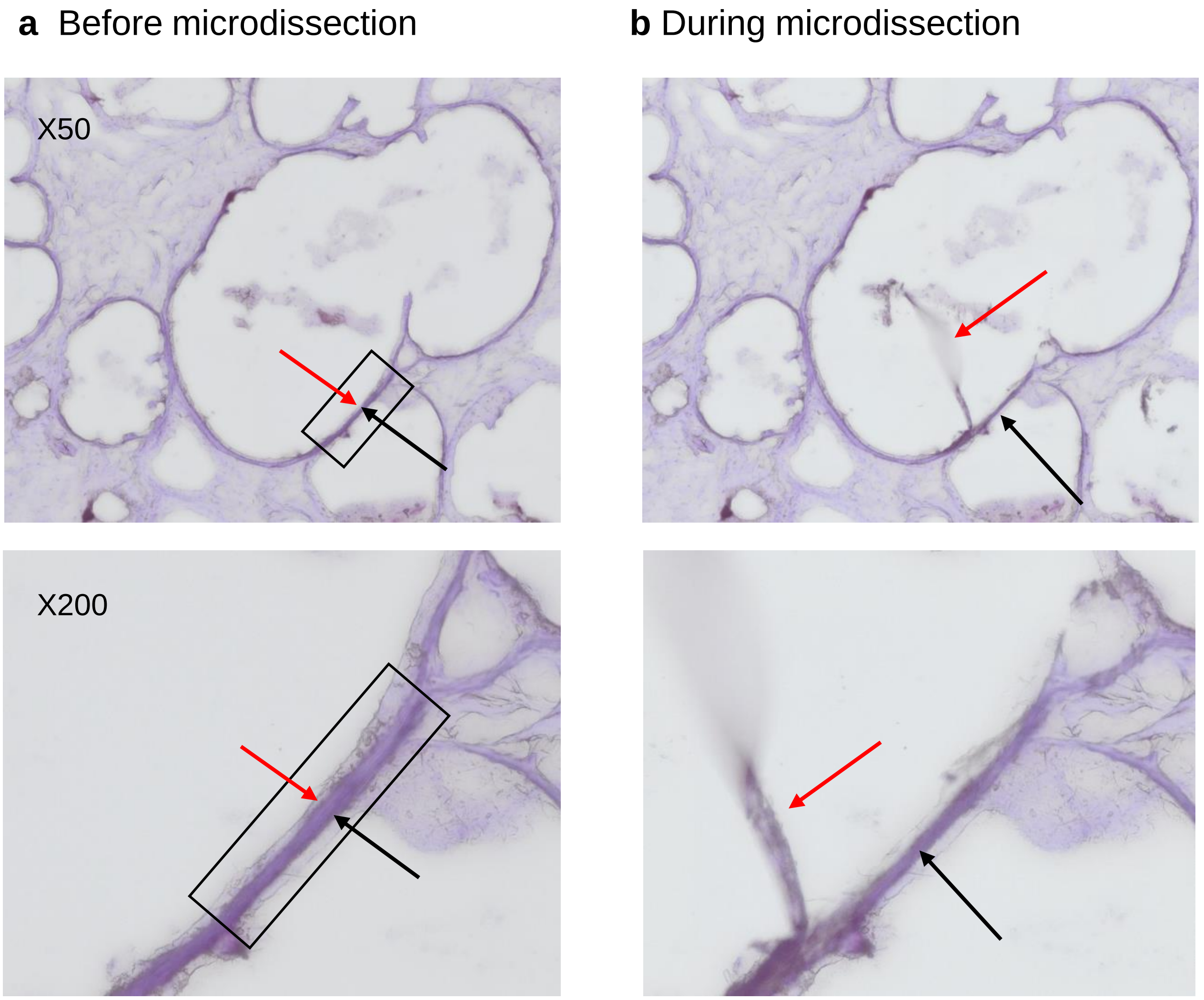

Supplementary Figure 2. Comparison of somatic profiles by histological types. a-b. Comparison with previous studies. The somatic single base substitution (SBS) burden (a) and estimated telomere length (b) analyzed by WGS are compared with those of previously published data. **a** An increase in the SBS burden during normal, benign prostatic hyperplasia (BPH), and prostate cancer (PCA) progression in the current study. Mutation burden of BPH samples from Liu *et al.* was extrapolated from whole-exome sequencing. **b** A decrease in the telomere length during normal, BPH, and PCA progression in the current study. PCAWG: pan cancer analysis of whole genomes, PRAD: prostate adenocarcinoma. **c** Number of structural variation is higher in PCA than normal and BPH. **d** Fraction of genome altered is higher in PCA than normal and BPH. Age-corrected linear regression analysis was performed.

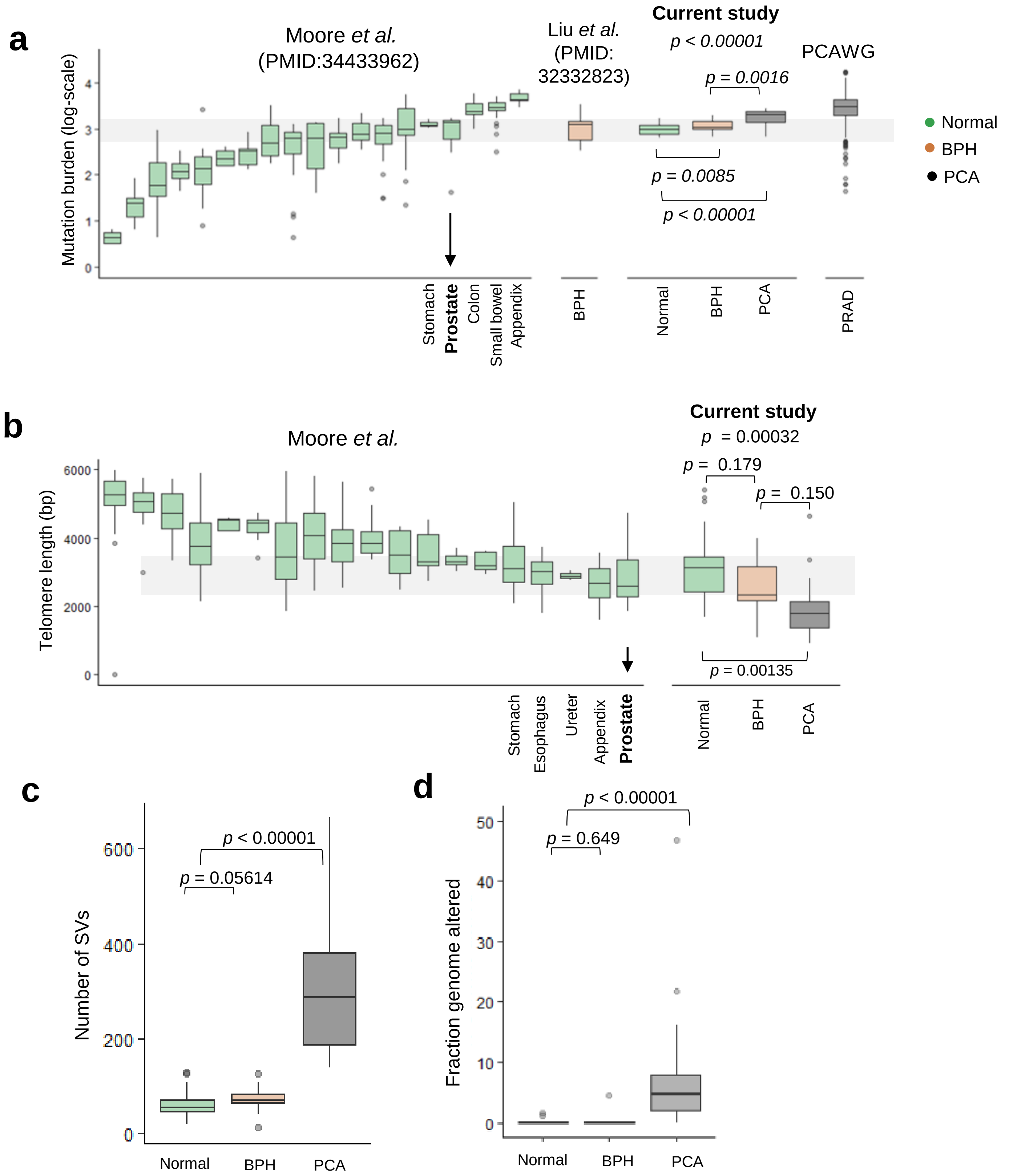

Supplementary Figure 3. Genomic profiles in two prostates with spatial 3D sequencing. a The somatic single base substitution (SBS) burden and estimated telomere length in multi-region sampling cases (PCA-28 and PCA-49) showing anti-correlation. **b** Mutational signatures of PCA-28 and PCA-49. Each bar represents each sample. **c** Proportion of APOBEC and ROS signatures are not different between normal and BPH samples in the PCA-28.

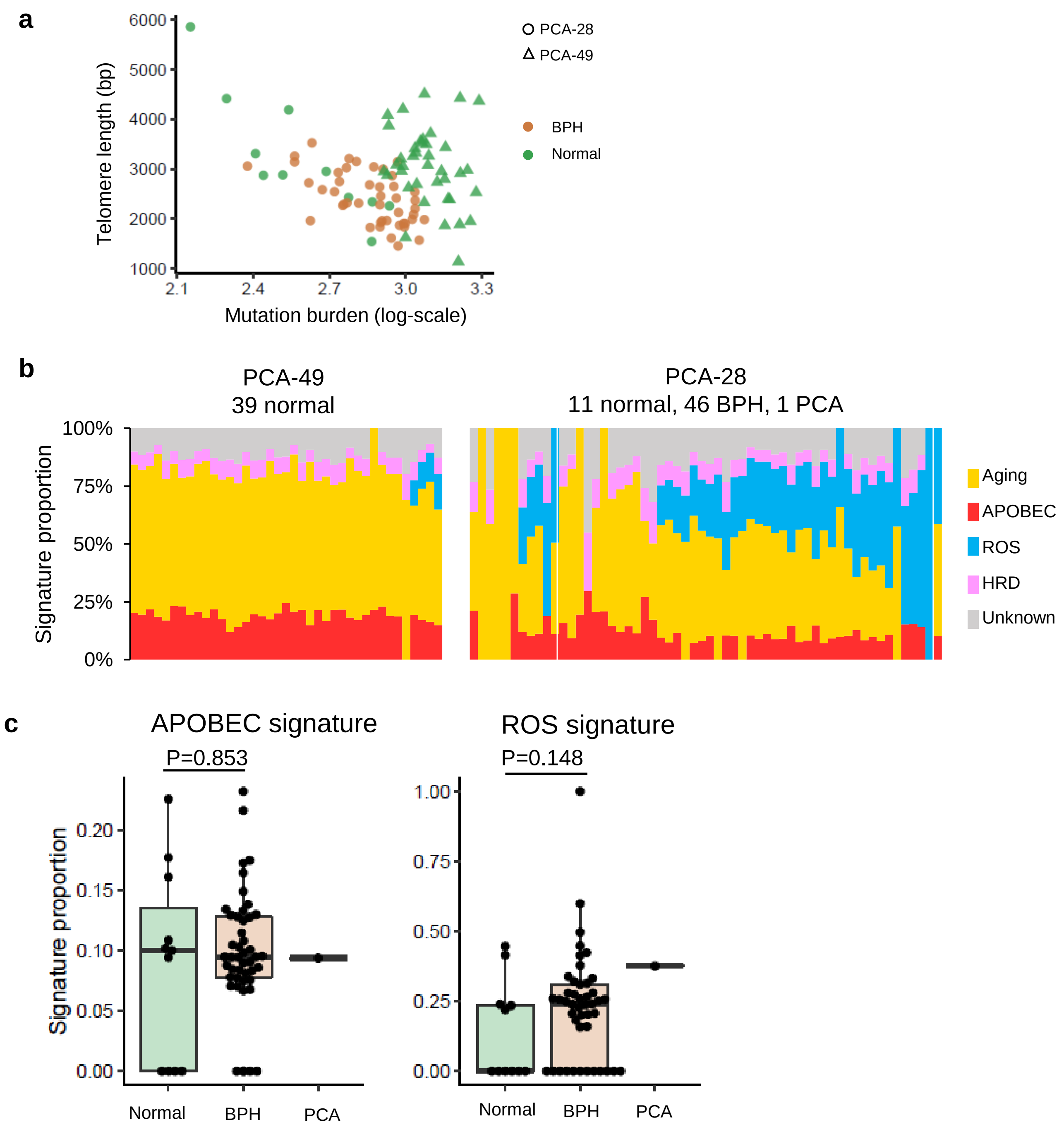

Supplementary Figure 4. Clustered genomic rearrangement events. Each structural variant cluster is shown with different color (innermost circos plot). Inter-mutation distance and base changes are displayed in the middle panel. 100kb-bin average coverage is shown in the outer circle. SV clusters are detected only in PCA clones.

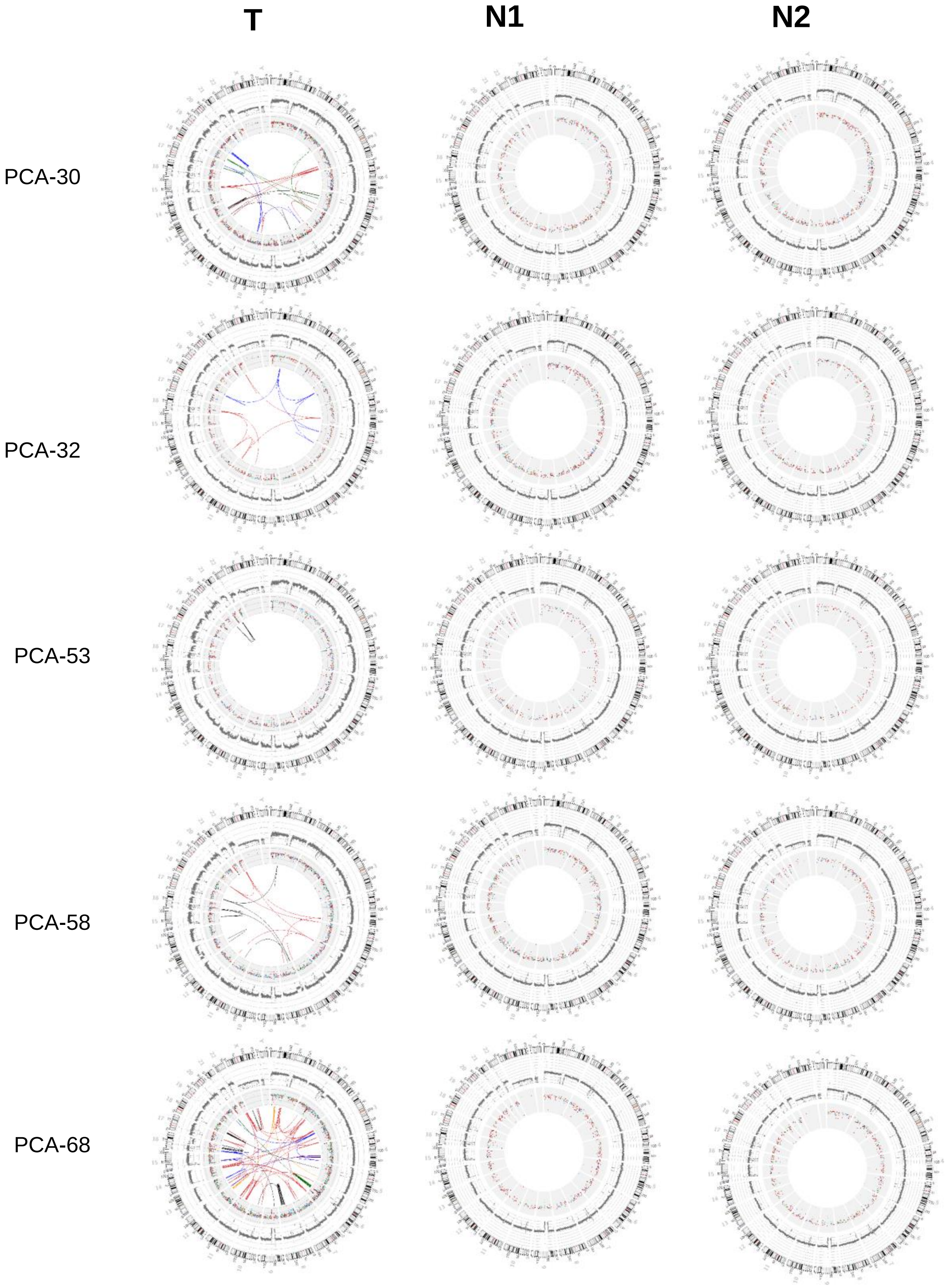

Supplementary Figure 4 (Continued)

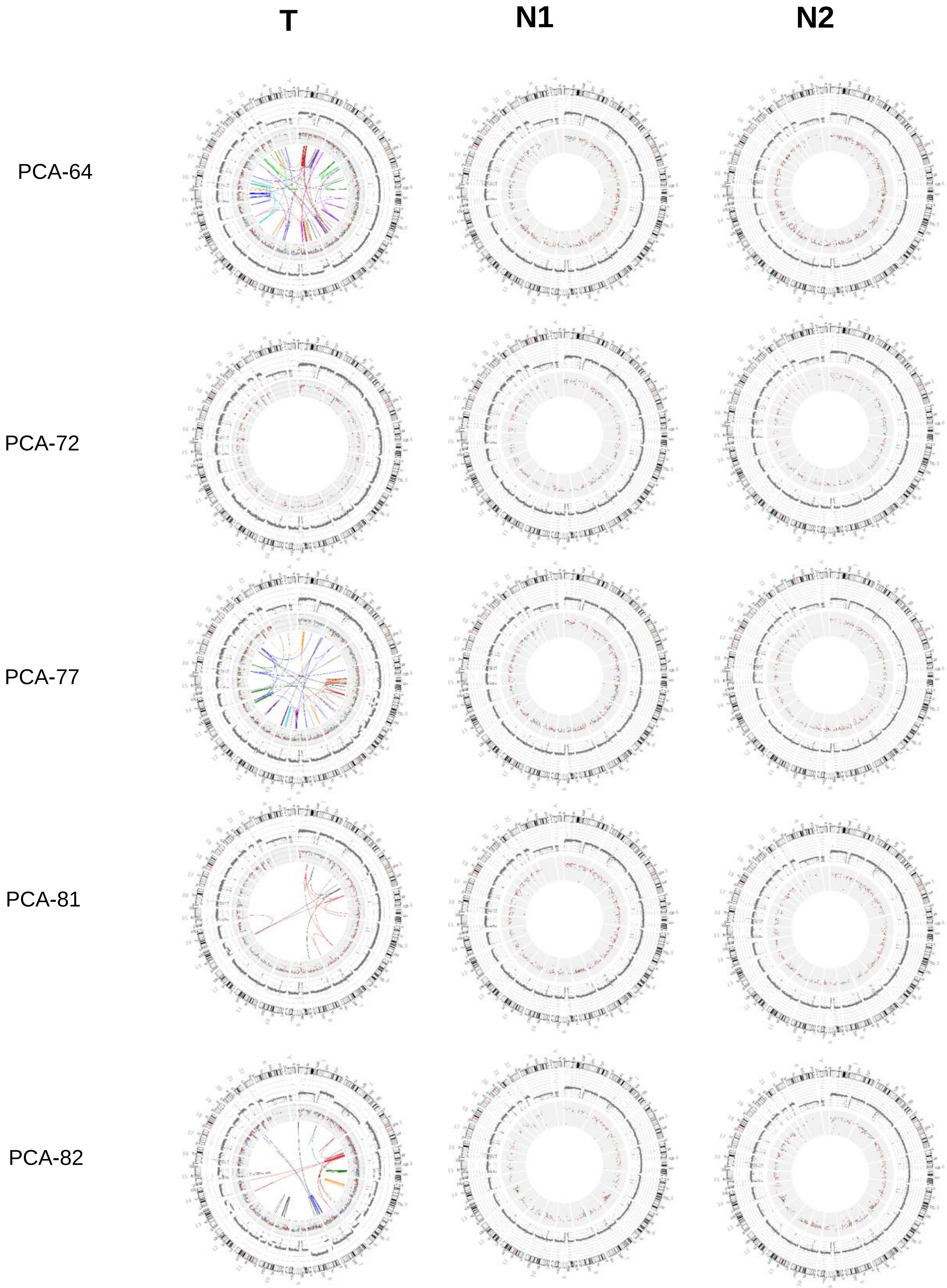

Supplementary Figure 4 (Continued)

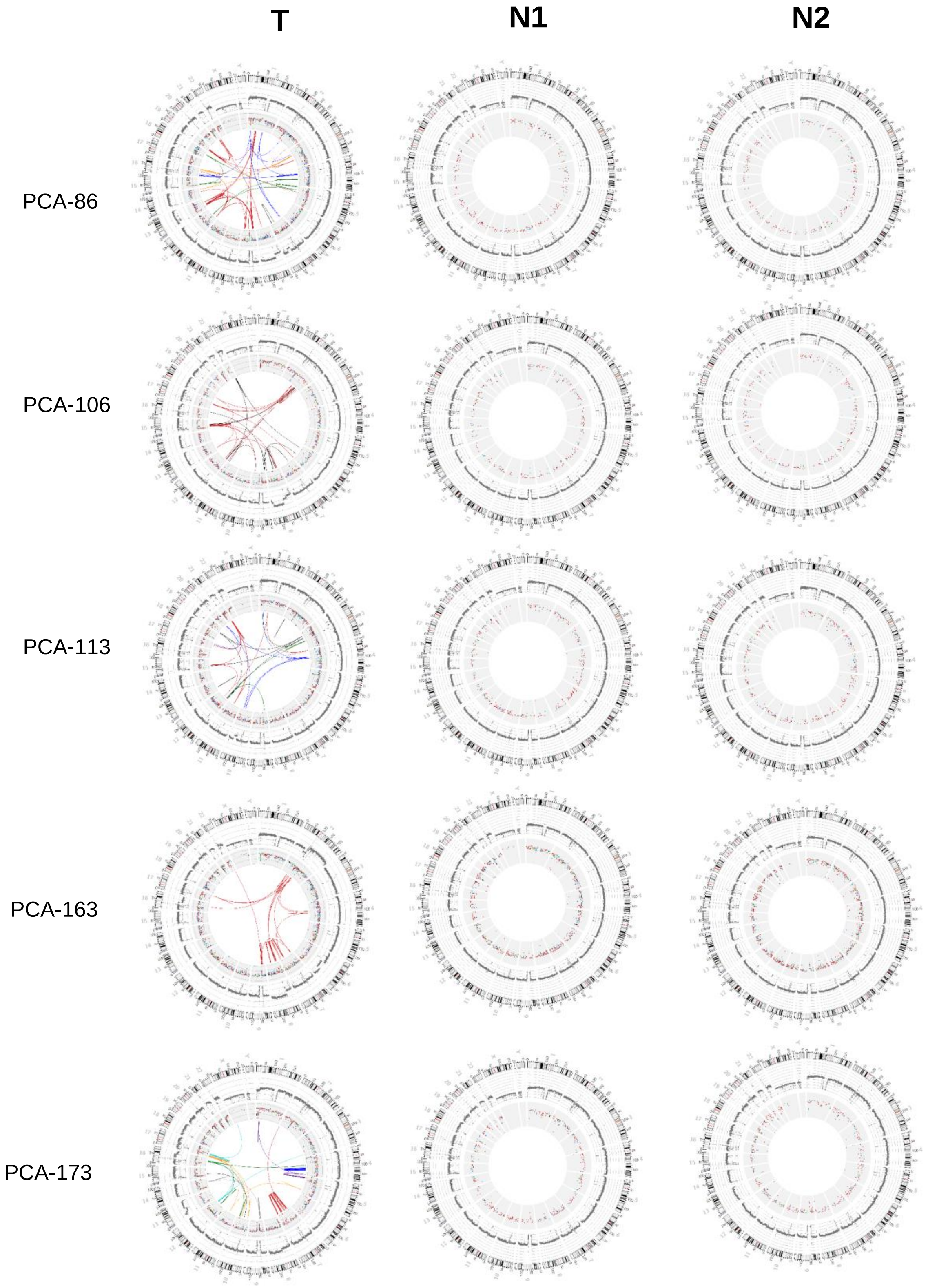

Supplementary Figure 4 (Continued)

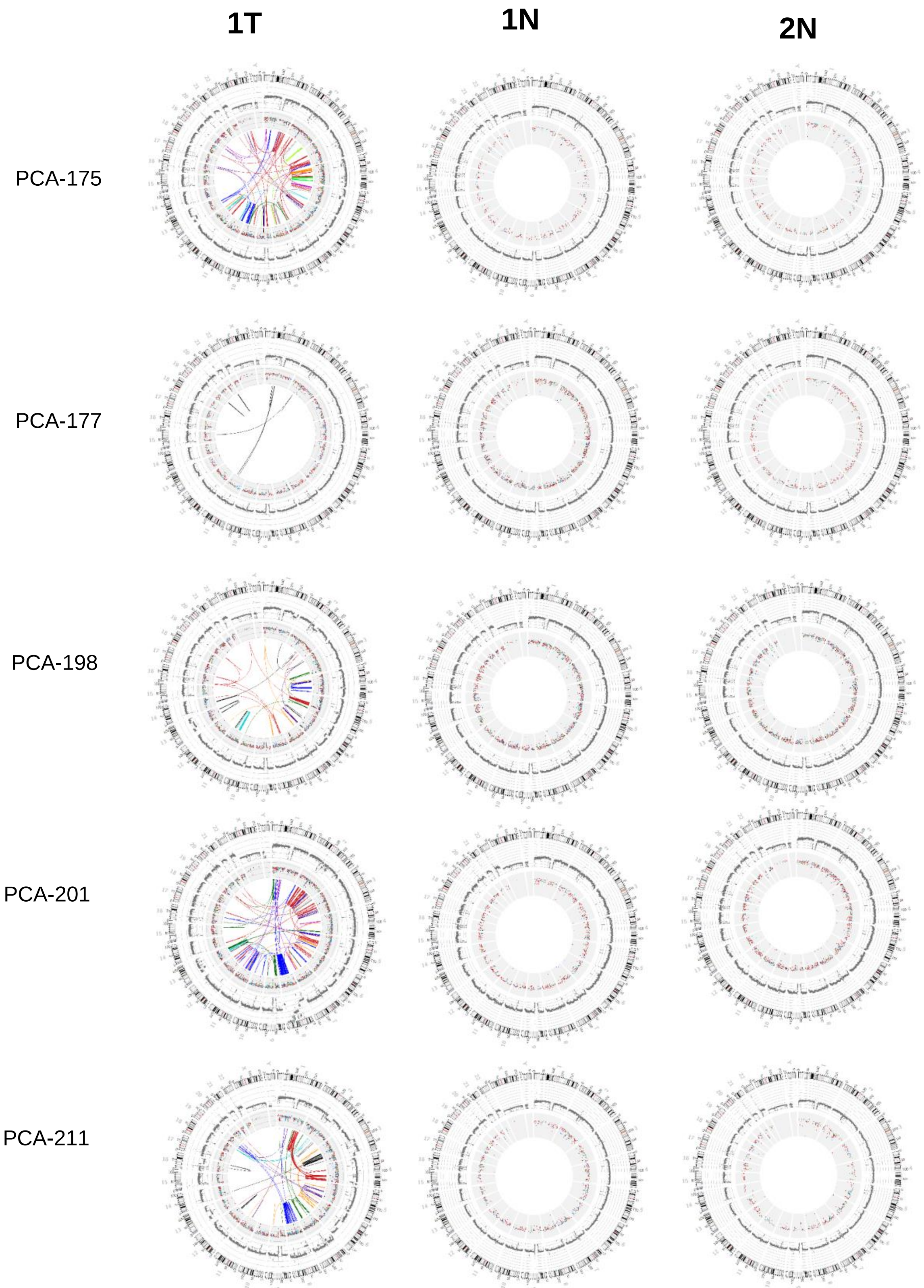

Supplementary Figure 5. Somatic copy number alterations in normal samples. x-axis: genomic position in bp. Blue: loss. absCN: absolute copy number estimated.

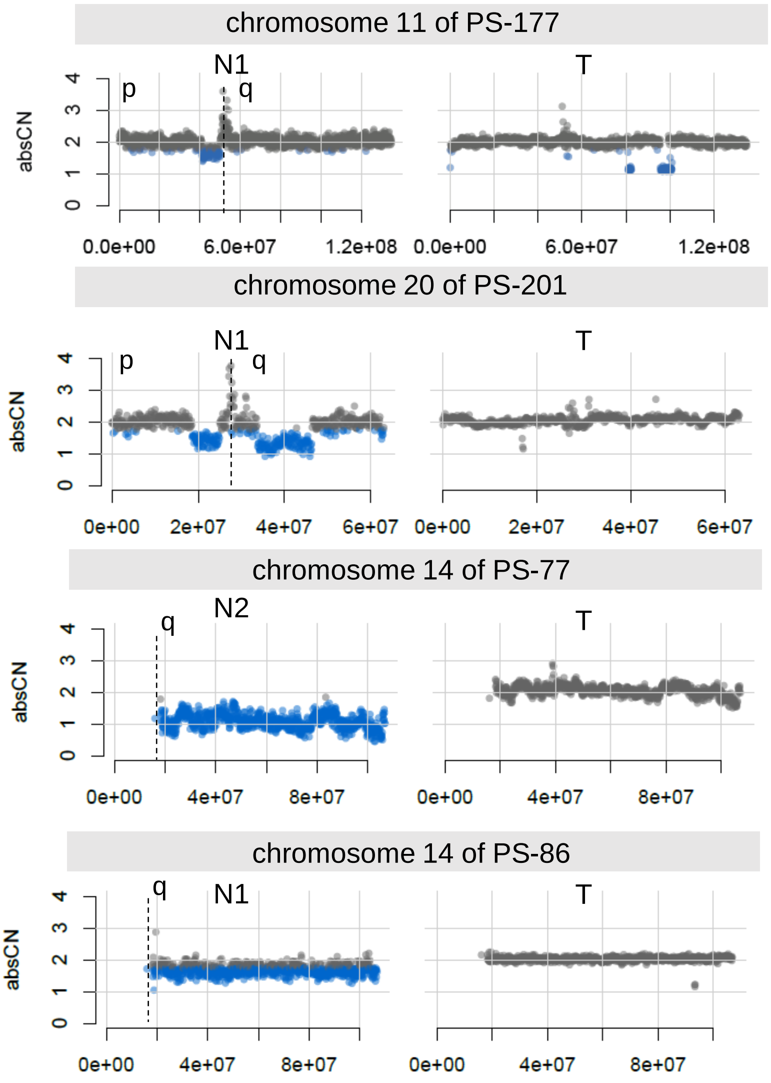

Supplementary Figure 6. Mutation burden and telomere length by tumor driver types. **a** Distribution in PCA samples. PCA samples with mutation-type driver (SPOP and FOXA1) show higher mutation burden and lower telomere length, while ETS fusion positive PCA samples show lower burden and longer telomere. **b** NSMs compared with corresponding tumor driver types. Tumor drivers are not related to NSM burden. Green: Normal. Brown: BPH.

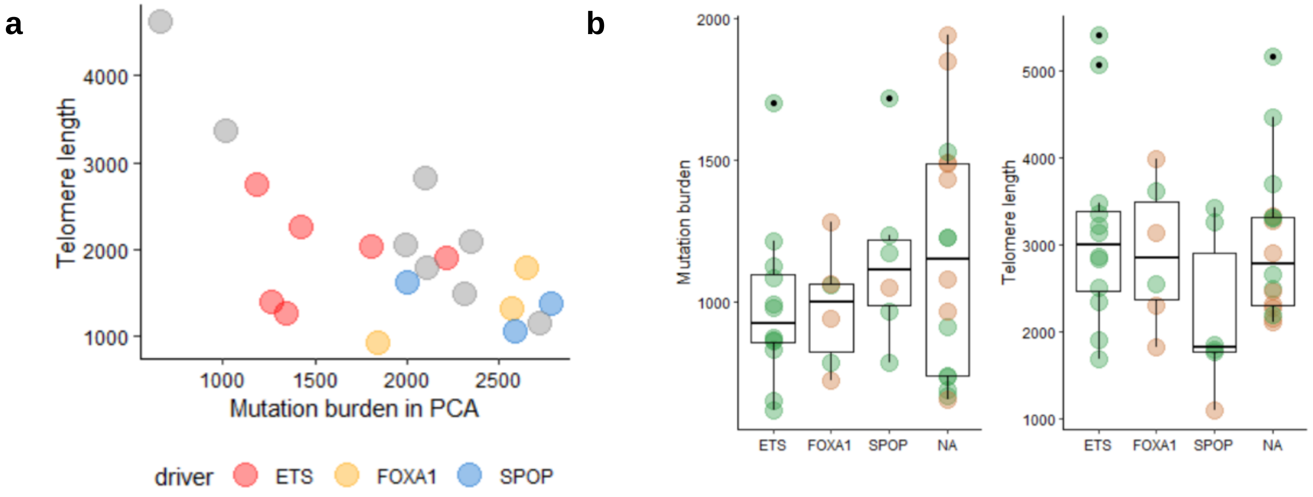

Supplementary Figure 7. Comparison of genomic profiles of BPH coexisting with prostate cancer to those of BPH without associated prostate cancer 9pure BPH). **a** Mutation burden significantly correlates with patients' ages (R = 0.55 and p = 0.008) and telomere lengths (R = -0.68 and p = 0.0004) . **b** Frequencies of driver mutation in COSMIC genes and those in normal tissue driver genes are similar between two BPH groups.

a

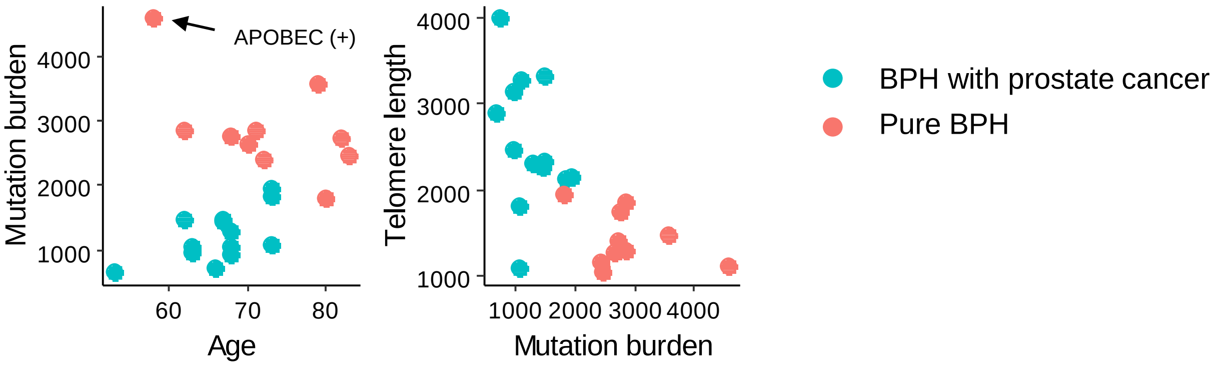

b

	**BPH with cancer** vs **pure BPH**	***P***
Somatic driver (COSMIC)	85% < 70%	n.s.
Somatic driver (PMID:36162751)	38% < 30%	n.s.

Supplementary Figure 8. Distribution of mutation allele frequency by mutation clusters. Mutations are categorized into four classes regarding sharedness across clones and clonality; Shared or private, and clonal or subclonal. **a** Six ETS fusion-positive PCAs. **b** Three SPOP mutated PCAs. **c** Six FOXA1 or FOXP1 mutated PCAs. **d** Five PCA without known cancer drivers.

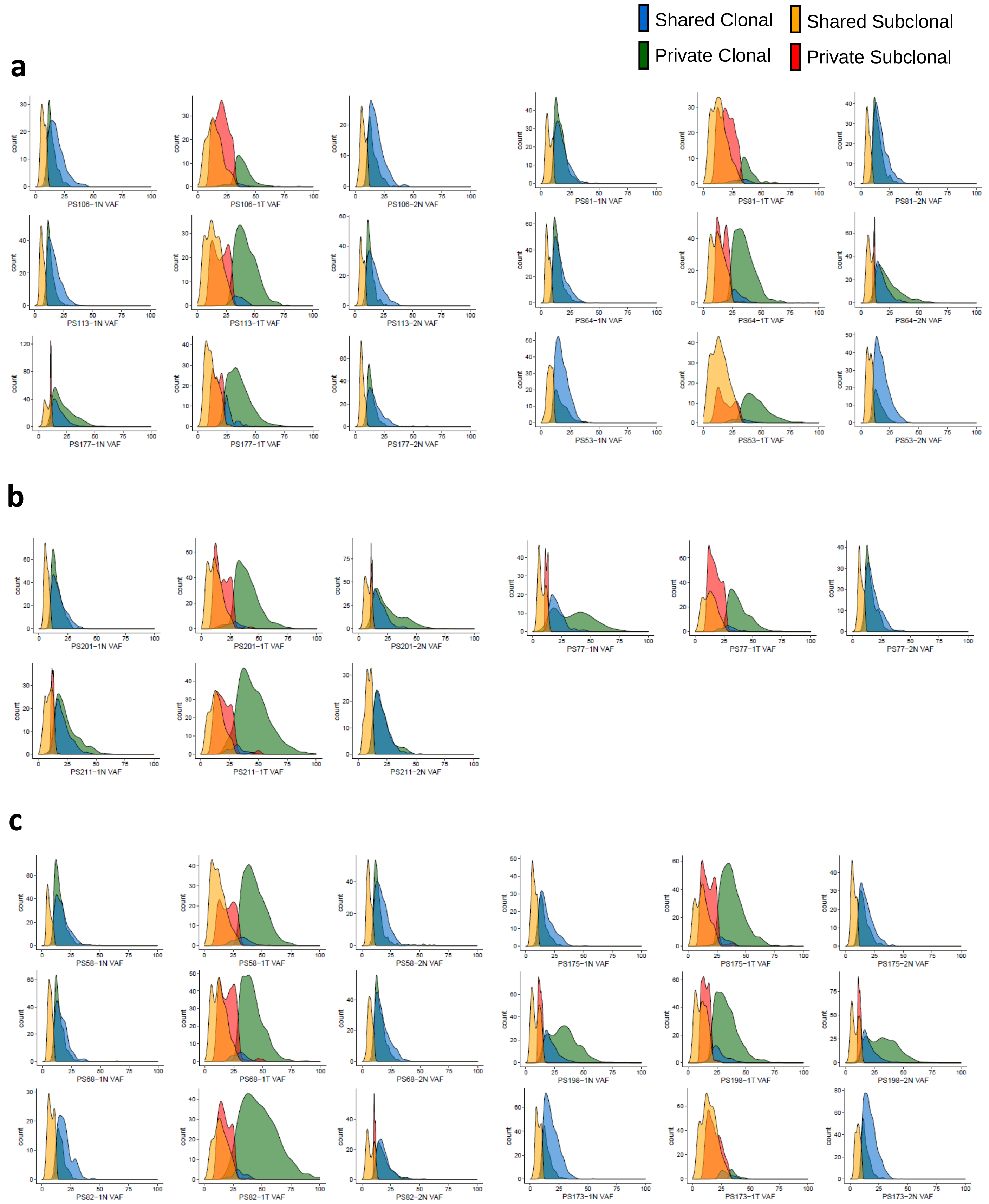

Supplementary Figure 8 (continued)

d

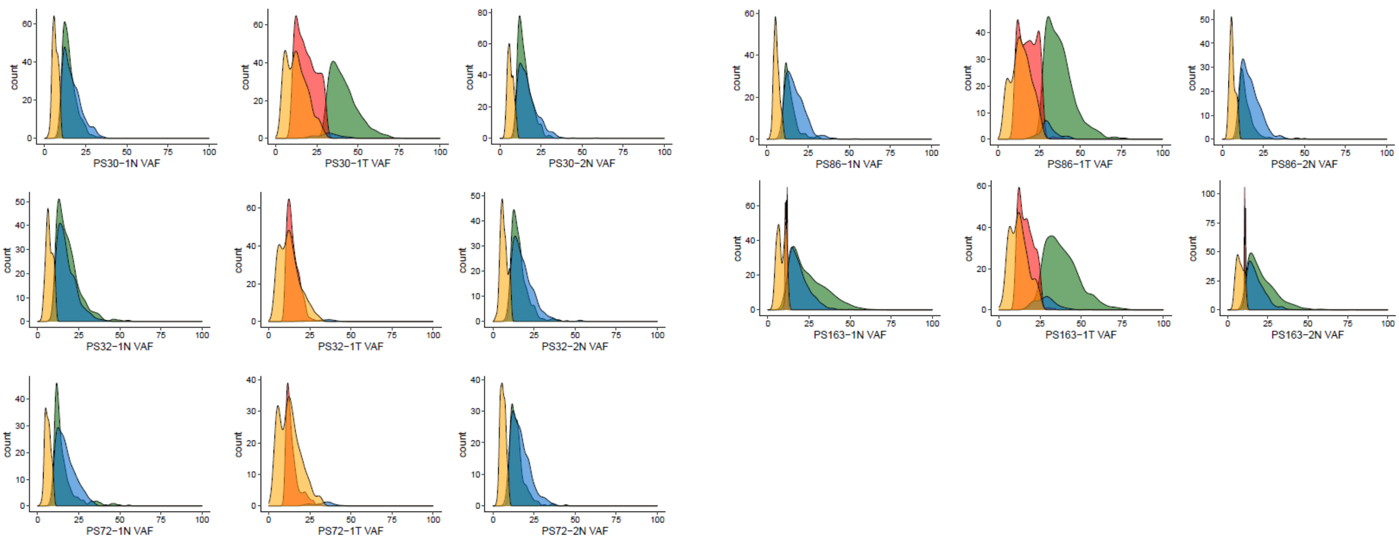

Supplementary Figure 9. Private clonal BCOR indels found in BPH regions of PCA-198.

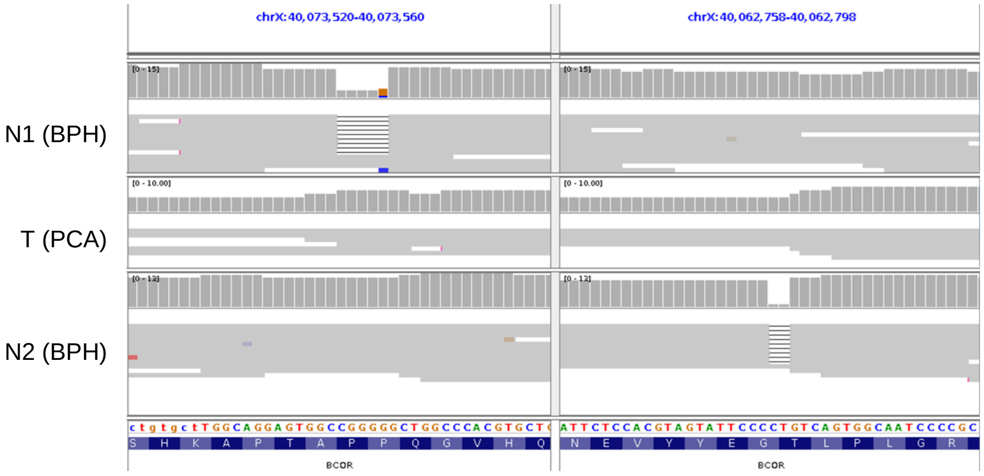

Supplementary Figure 10. Phylogenetic trees reconstructed with somatic mutations. Each phylogenetic tree of three clones including PCA clone is constructed with maximum likelihood algorithm of MegaX program. Age of diagnosis and major driver alteration of PCA clone are shown. 1N: PCA-close normal or BPH. 1T: PCA. 2N: PCA-away normal or BPH. BPH is shown with an orange dot.

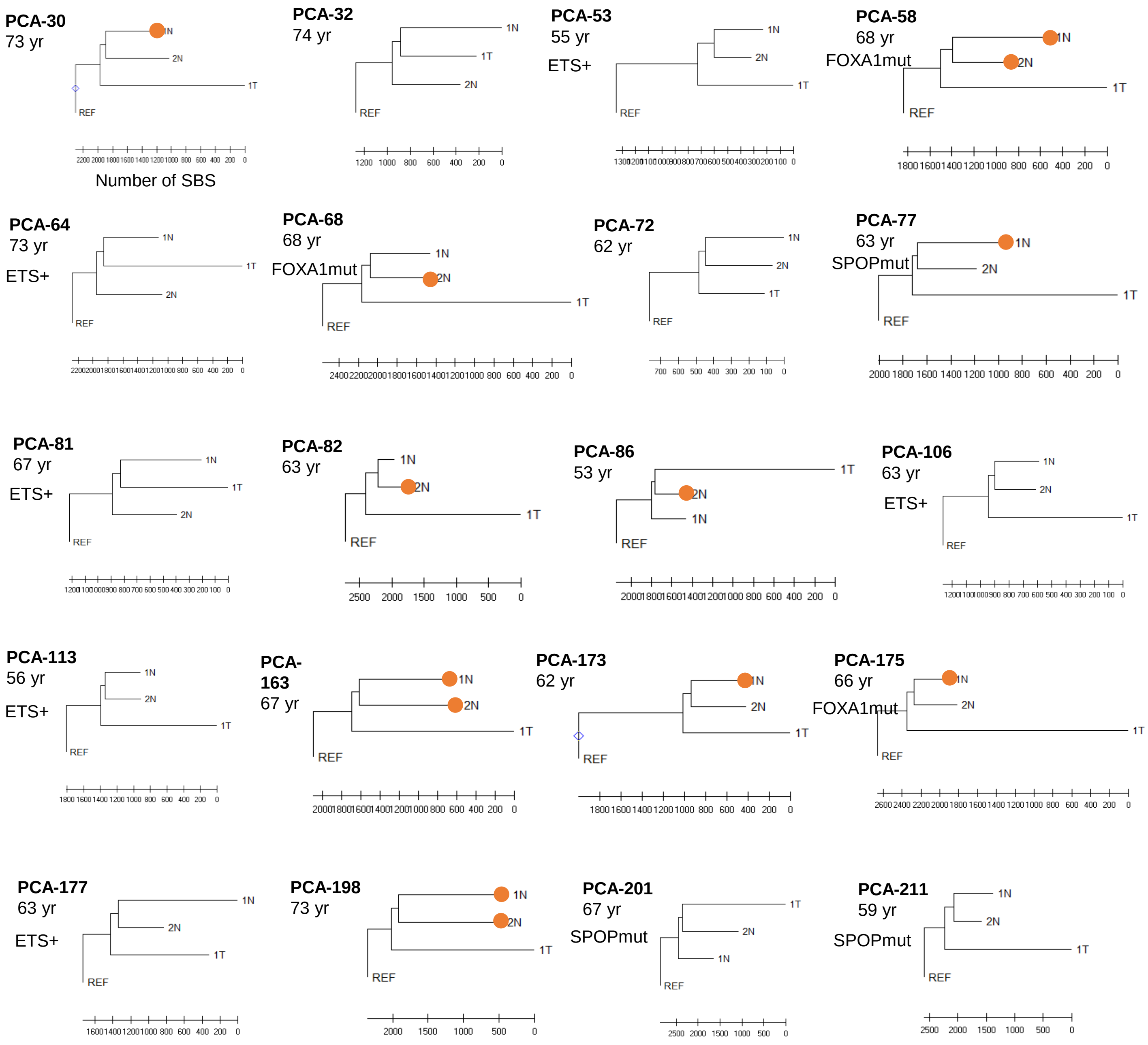

Supplementary Figure 11. Contribution of aging and ROS signatures in PC and SC clusters in PCA samples. Private clonal (PC) mutations of PCA samples showed a significant level of ROS signature.

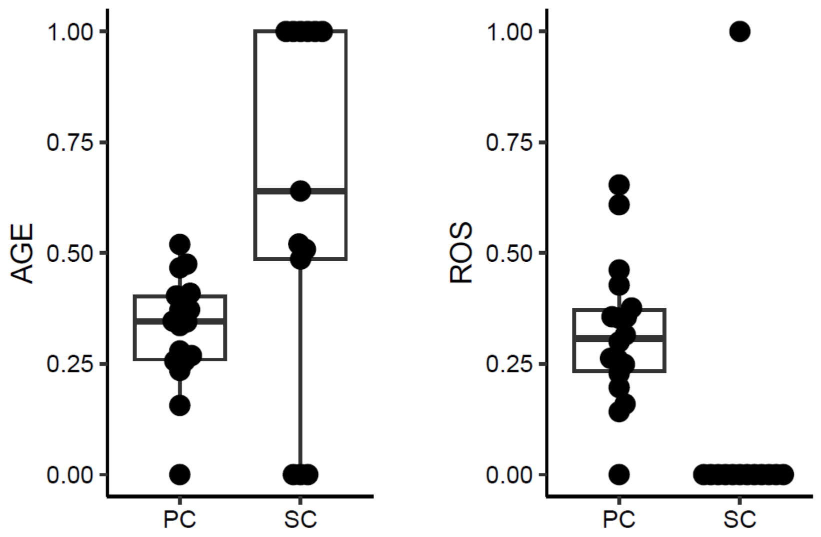

Supplementary Figure 12. Dissection and pathologic review of two spatial sequencing cases. a58 areas sampled and sequenced in PCA-28. **b** 48 areas sampled and sequenced, and 39 areas used for analysis in PCA-49.

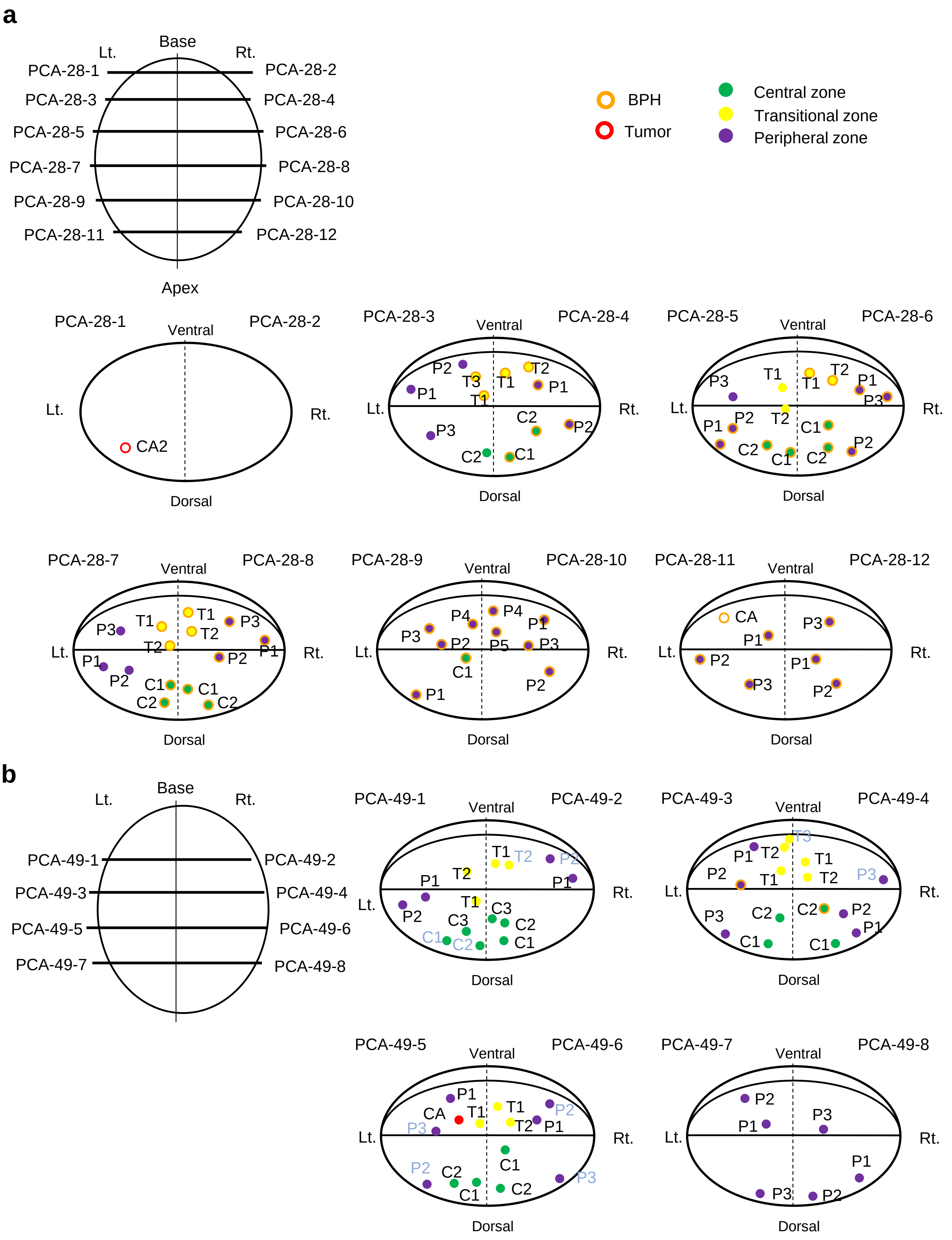

Supplementary Figure 13. Sidedness of genomic profiles in two multi-region sequencing cases. a-b Mutation burden and telomere lengths are compared by left/right, upper/lower, and anterior/posterior axis. **c** Comparison of the number of private mutations without early mutations in PCA-28. Private somatic mutations shows slight right-side enrichment in BPH regions of PCA-28. **d** Left/right distribution of somatic mutations and telomere lengths by pathologic zones in BPH regions of PCA-28 case. Left-side: green. Right-side: red. N.S.: not significant.

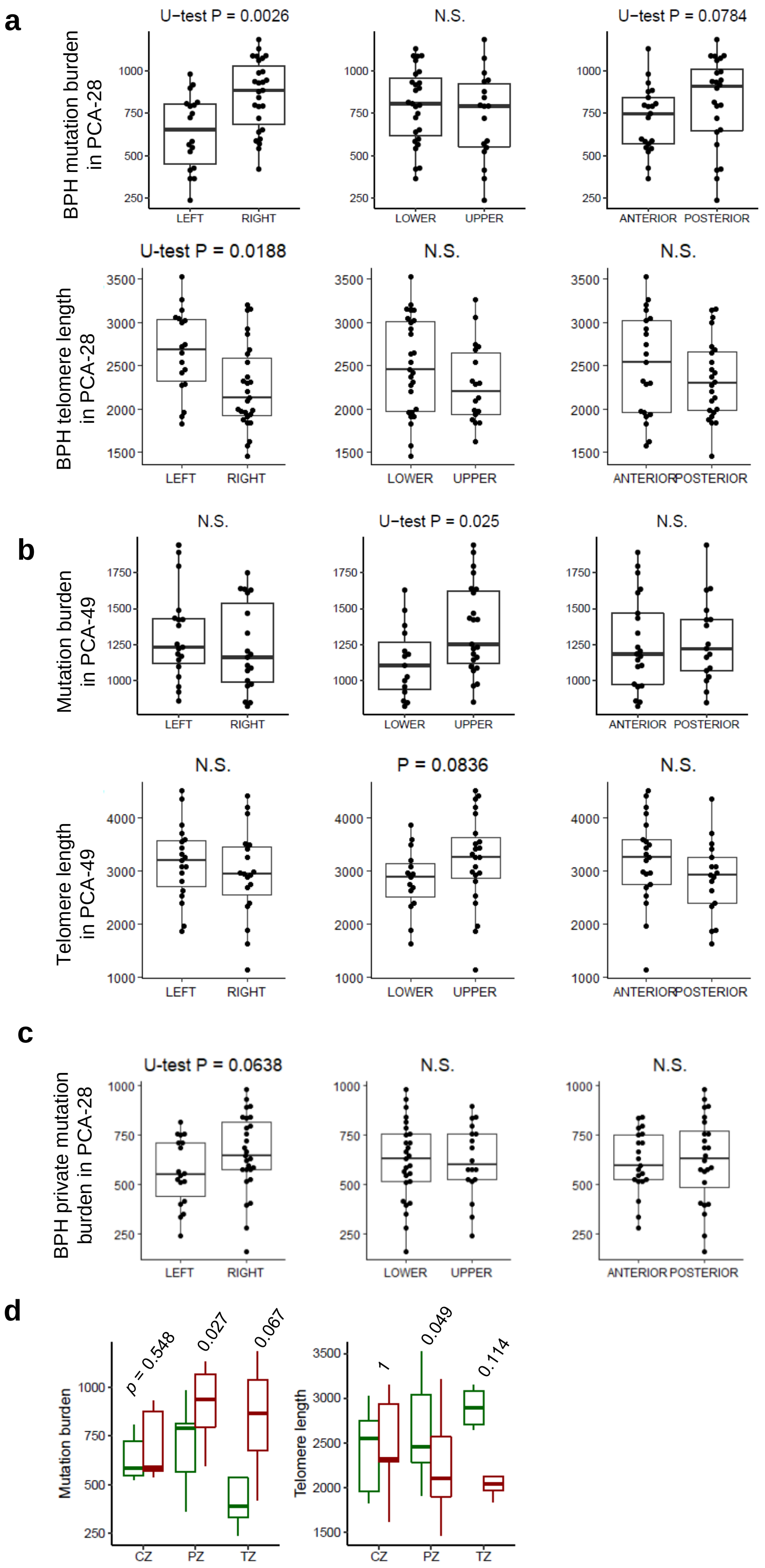

Supplementary Figure 14. Somatic mutations on RAS-RTK pathway. a Somatic mutations on RAS-RTK pathway and mutated samples. Genes listed in KEGG-Ras signaling pathway and REACTOME-Signaling by receptor tyrosine kinases were used. **b** Proportion of samples with RAS-RTK pathway gene mutations. **c** Allele frequency comparison of cancer hot-spot mutations in panel sequencing and WGS.

a

refGene	AAChange	OncoKB	Sample_Barcode	type
SH2B3	p.R83W	Unknown	PCA-113-1T	PCA
PTPRZ1	p.G1294Efs*16	Unknown	PCA-175-1T	PCA
KIDINS220	p.D437Vfs*45	Unknown	PCA-198-1N	BPH
WWP1	p.G16E	Unknown	PCA-201-1N	Normal
HSP90AA1	p.V597Gfs*23	Unknown	PCA-211-1T	PCA
ATP6V0A4	p.R663Efs*100	Unknown	PCA-58-1N	BPH
INSR	p.T68M	Unknown	PCA-58-2N	BPH
STK4	p.R12Q	Unknown	PCA-64-1T	PCA
ROCK1	p.G88V	Unknown	PCA-82-2N	BPH
VAV3	p.G405D	Unknown	PCA-86-1T	PCA
NFKB1	p.R192Gfs*5	Unknown	PCA-86-1T	PCA
ALK	p.V324I	Unknown	PCA-28-10-P5	BPH
GFAP	p.G32S	Unknown	PCA-28-3-P2	Normal
EPS15L1	p.V294M	Unknown	PCA-28-4-P1	BPH
PIK3R1	p.E52K	Unknown	PCA-28-4-P1	BPH
TNS3	p.S332A	Unknown	PCA-28-4-P1	BPH
NCOR1	p.E533K	Unknown	PCA-28-9-P1	BPH
BRAF	p.K601E	Likely Oncogenic	PCA-49-1-P2	Normal
ERBB2	p.R678Q	Oncogenic	PCA-49-4-P2	Normal
NCKAP1L	p.Q376E	Unknown	PCA-49-4-T1	Normal
CHD4	p.R1619S	Unknown	PCA-49-5-CA	Normal
RASA1	p.N322H	Unknown	PCA-49-5-CA	Normal
GABRG2	p.Y122X	Unknown	PCA-49-5-CA	Normal
RRAS2	p.Q37L	Oncogenic	PCA-49-6-C1	Normal
PIK3CA	p.E80K	Unknown	PCA-49-6-C1	Normal
HRAS	p.Q61R	Likely Oncogenic	PCA-49-8-P2	Normal
CHD4	p.D1727Y	Unknown	PCA-49-8-P3	Normal

b

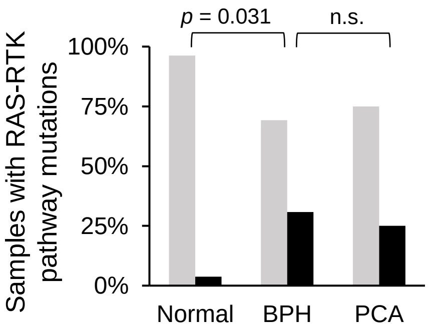

C

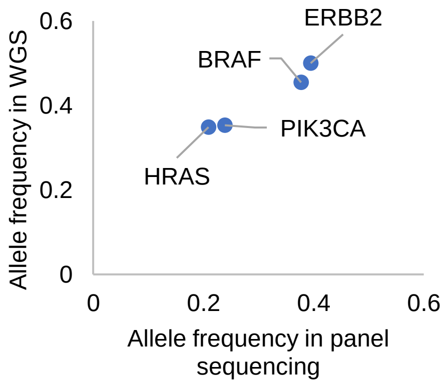

Supplementary Figure 15. Mutation burdens and telomere lengths by tumor proximity. a Significant differences in mutation burden and telomere length are not observed between PCA-close (N1) and PCA-away (N2) areas. **b** Zonal difference in normal regions is not found. Most of BPH regions were in PZ. **c** Ratios of mutation burden and telomere length for matched N1 and N2 show significant difference. Linear regression considering age as a covariate was performed in (a-b). Paired T-test was performed in (c).

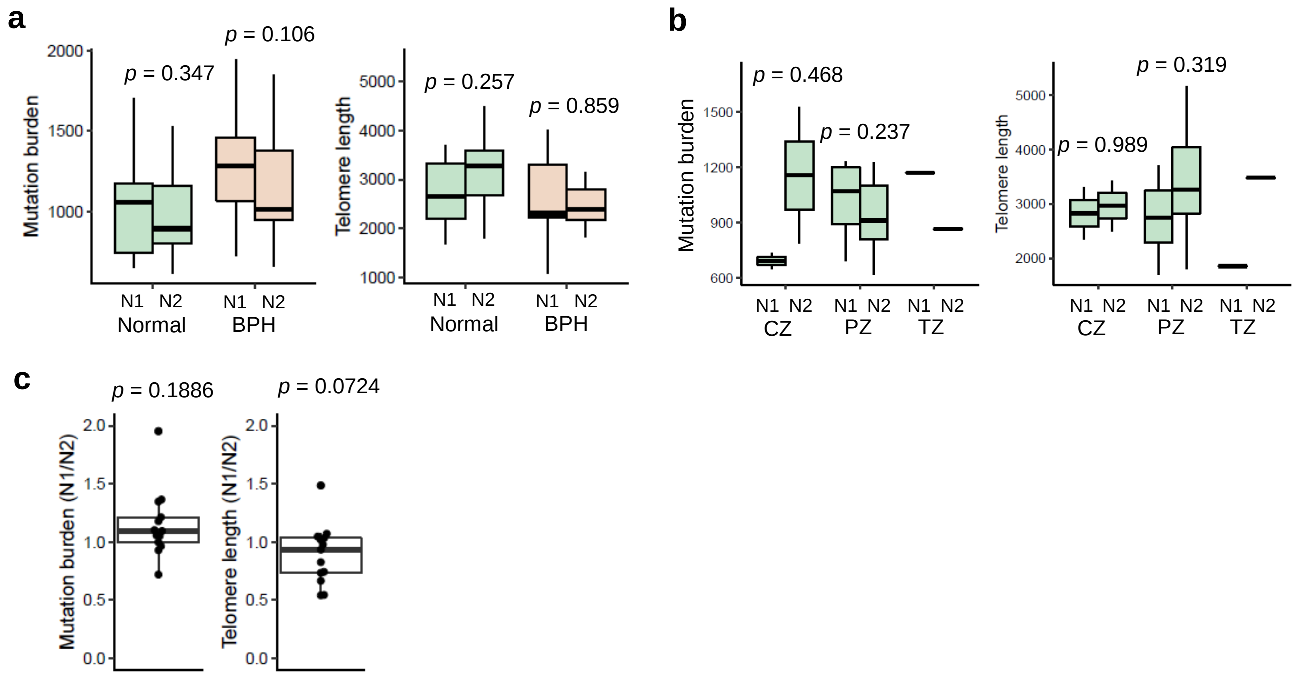

Supplementary Tables

Supplementary Table 1. Clinicopathological features of 22 PCA patients used in the study

Supplementary Table 2. Summary of whole genome sequencing of 20 patients with trio sampling

Supplementary Table 3. Summary of WGS of PCA-28. n.p.: not performed.

Supplementary Table 4. Summary of WGS of PCA-49

Supplementary Table 5. Amplicon panel target information (hg19)

Supplementary Table 6. Somatic nonsilent mutations of normal and BPH samples found in deep panel sequencing (hg19)

Supplementary Table 7. List of somatic non-silent mutations in coding regions found in normal and BPH samples by WGS (hg38)

Supplementary Table 8. Somatic structural variants discovered by WGS

Supplementary Table 9. Percentage of copy number alteration (CNA) identified by WGS

Supplementary Table 10. Clinical characteristics and mutation burden. For each clinical features, correlation coefficient with mutation burden and significance are shown.

Supplementary Table 11. Summary statistics of 10 BPH patients without prostate cancer.

Supplementary Table 12 List of somatic non-silent mutations in coding regions found in pure BPH samples by WGS (hg38)
